# Integrated metagenomic and metabolomic analysis identifies severity-specific inflammatory and metabolic signatures in post-stroke depression

**DOI:** 10.1080/19490976.2026.2726620

**Published:** 2026-09-14

**Authors:** Wenyi Chen, Yawen Pan, Mengyuan Chen, Shanshan Zhou, Xiaodie Liu, Mengjiao Sun, Zixuan Yang, Yinghao Zhi

**Affiliations:** a Wenzhou Joint Training Base, Zhejiang Chinese Medical University, Wenzhou, China; b Department of Rehabilitation Medicine, The Second People's Hospital of Lishui, Lishui, China; c Department of Rehabilitation Medicine, Wenzhou TCM Hospital of Zhejiang Chinese Medical University, Wenzhou, China

**Keywords:** Post-stroke depression, gut microbiota, metabolomics, multi-omics integration, inflammation, biomarkers

## Abstract

Post-stroke depression (PSD) is a common complication that significantly impacts patient prognosis. This study aimed to systematically characterize the associations among gut microbial ecology, metabolic profiles, and inflammatory responses across different severities of PSD. We conducted metagenomic sequencing, non-targeted metabolomics, and serum cytokine analysis (IL-1β, IL-6, IL-10, IL-18, TNF-*α*, IFN-*γ*, and CRP) in 91 patients with varying degrees of PSD and non-PSD controls. Bioinformatics analyzes were employed to construct multi-omics association networks and machine learning models. Results indicated that PSD patients exhibited significantly increased gut microbiota alpha-diversity, suggesting dysbiosis. Mild depression was characterized by compensatory neural signaling activation, whereas the moderate depression group exhibited abnormalities in tryptophan/indole metabolism, oxidative stress-related metabolic imbalances, and functional decompensation. Further analyzes suggested that *Alistipes*, *Blautia_A*, *Evtepia gabavorous*, and *Lachnospira* were associated with inflammatory features, GABA-related metabolic alterations, aromatic amino acid/indole metabolism, and lipid-amino acid metabolism, respectively. Under a more rigorous 10-fold cross-validation framework, the performance of different multi-omics combination models showed heterogeneity; however, some combinations still demonstrated superior discriminatory ability compared to single-omics approaches. This study provides multi-omics clues suggesting associations between different PSD severity levels and features such as increased *Alistipes* abundance, reduced antioxidant capacity, and altered tryptophan metabolism. It provides candidate biomarker combinations that may be useful for PSD stratification and suggests that the gut microbiome may represent a potential target for future PSD intervention. In summary, PSD may be associated with dynamic alterations along the “gut–brain-inflammation-metabolism” axis. These findings provide integrated evidence for microbial, metabolic, and inflammatory abnormalities across different PSD severity levels, but still require validation in larger samples, longitudinal cohorts, and mechanistic studies.

## Graphical abstract

**Figure:**
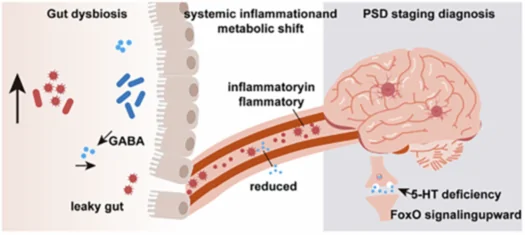

## 1. Introduction

Post-stroke depression (PSD) is a prevalent neuropsychiatric complication affecting approximately one-third of stroke survivors.^1^ Characterized by persistent anhedonia and despair, PSD significantly impairs neurological recovery, cognitive rehabilitation, and quality of life, while increasing mortality risk.^2^ Currently, diagnosis relies on subjective questionnaire-based assessments, which lack specificity and sensitivity. Although existing research has identified mechanisms such as inflammatory responses, neurotransmitter imbalances, and hypothalamic-pituitary-adrenal (HPA) axis dysfunction contributing to PSD development, its precise pathophysiological mechanisms remain incompletely understood. Currently, diagnosis relies on subjective questionnaire-based assessments, which lack specificity and sensitivity.

Recently, the “microbiome–gut–brain axis” has offered a novel framework for elucidating PSD mechanisms. Gut microbiota modulate CNS function via immune regulation, energy metabolism, and neurotransmitter synthesis. Studies reveal that PSD patients exhibit significant dysbiosis, characterized by altered Firmicutes/Bacteroidetes ratios, reduced butyrate producers, and increased opportunistic pathogens. This dysbiosis impairs the intestinal barrier, facilitating the translocation of pro-inflammatory factors into the circulation. This process activates neuroimmune responses and alters cortical gene expression,^3^ mediated by pathways such as vagus nerve signaling, tryptophan metabolism, and short-chain fatty acids (SCFAs).^4^ Existing research has further demonstrated that alterations in the gut microbiota are highly associated with host metabolic abnormalities. In multi-omics integrated analyzes of patients with PSD, depression, and stroke, the gut microbiota has been shown to regulate pathways related to energy metabolism, inflammatory responses, and neurotransmitter synthesis, influencing serum and fecal metabolic characteristics.^5^ Correlation analysis between the microbiome and metabolomics has been widely adopted as an effective strategy for understanding the relationship between health outcomes and the microbiome.^6^ Hernández-Cacho et al. utilized integrated analysis of fecal metabolomics and microbiome data, revealing significant alterations in specific bacterial genera (such as Acidaminococcus and Megasphaera) and associated lipid and organic acid metabolites in depressed patients, suggesting the critical role of microbiota-metabolism interactions in depression pathogenesis.^7^ These findings provide important references for exploring the microbial-metabolic mechanisms of PSD.

However, current multi-omics studies on gut microbiota, fecal metabolism, and serum metabolism remain limited, particularly lacking research comparing microbiota structure and metabolic phenotypes across different degrees of depression. Therefore, this study conducted gut metagenomic sequencing, non-targeted metabolomic analysis of fecal and serum samples, correlation analyzes between metagenomics and metabolomics, and inflammatory cytokine testing. The aim was to identify the gut microbial and metabolic characteristics of PSD patients across different severity levels of depression, explore candidate biomarkers associated with disease stratification, and provide insights for subsequent mechanistic studies and clinical identification.

## 2. Materials and methods

### 2.1. Study population

This study enrolled 91 stroke patients meeting inclusion criteria who were treated at the Rehabilitation Department or Neurology Outpatient/Inpatient Units of Wenzhou Hospital of Traditional Chinese Medicine, affiliated with Zhejiang University of Chinese Medicine, between January 2023 and December 2024. The cohort comprised 33 patients without post-stroke depression (NPSD group) and 58 patients with post-stroke depression. To investigate disease progression, patients with post-stroke depression were further categorized into mild (HAMD score 8–17, *n* = 31) and moderate (HAMD score 18–24, *n* = 27) groups. Matched fecal and blood samples were collected from all participants for metagenomic and metabolomic analysis. Fresh stool samples were collected and stored at −80 °C until fecal DNA extraction. Blood samples (5 mL) were collected the morning after stool collection following an overnight fast. Samples were allowed to clot at room temperature for 30 minutes, then centrifuged at 2000 × g for 15 minutes to separate serum. The resulting supernatant was aliquoted and stored at −80 °C until testing.

Inclusion criteria included: age 18–80 y, with communication ability; meeting the diagnostic criteria for stroke imaging and the “Chinese Expert Consensus on Post-Stroke Depression (PSD)” (2016); and depression occurring within 2 weeks to 1 y after stroke. Exclusion criteria included severe aphasia or cognitive impairment, history of mental illness, suicidal tendencies, recent use of antibiotics/probiotics, gastrointestinal diseases that may affect gut microbiota, pregnancy, or serious systemic diseases. Dropout and exclusion criteria included adverse events, loss to follow-up, poor adherence, and incomplete data.

Ethical requirements and review number: Detailed communication with patients was conducted before enrollment, and informed consent was obtained. The clinical trial has passed the ethical review of the Wenzhou Municipal Hospital of Traditional Chinese Medicine, affiliated with Zhejiang University of Traditional Chinese Medicine (review number: WZY2023-LW-076-01). The clinical trial registration number is ChiCTR2300068128.

### 2.2. Fecal DNA extraction and metagenomic sequencing

Approximately 200 mg of fecal sample was collected immediately after defecation in the morning (from the middle section of the feces to reduce contamination). The sample was placed in a sterile centrifuge tube and stored at −80 °C, then transported to the experimental platform via a dry ice cold chain. All samples were numbered before the experiment to ensure blinding and traceability during the testing process. In total, 1 μg of qualified DNA was randomly fragmented using a Covaris ultrasonic disruptor, and target fragments of 200–400 bp were recovered by magnetic bead screening. The fragment-screened DNA underwent end repair, 3' A addition, and adapter ligation under suitable temperature conditions. The ligation products were amplified by PCR to enrich successfully ligated DNA fragments, and then purified by magnetic beads to obtain high-quality amplified fragments. The purified products were then denatured into single strands, and a single-stranded circular library was constructed in a circularization reaction system. Uncircularized linear molecules were removed by enzymatic digestion to improve library purity. The final library was sequenced using the DNBSEQ platform and PE150 (BGI Genomics Co., Ltd., China).

### 2.3. Sample preparation for non-targeted metabolomics of feces and serum

After slowly thawing the fecal sample at 4 °C, weigh 25 mg and place it in a 1.5 mL centrifuge tube. Add 800 μL of pre-cooled extraction buffer (methanol:acetonitrile: water = 2:2:1, v-v:v) and 10 μL of internal standard. Add two small steel balls and grind (50 Hz for 5 minutes). Sonicate in a 4 °C water bath for 10 minutes, then place in a −20 °C freezer for 1 hours. Subsequently, centrifuge the sample at 12,000 rpm at 4 °C for 15 minutes and collect 600 μL of supernatant. Dry the supernatant in a freeze concentrator, add 600 μL of reconstitution solution (methanol: H2O = 1:9, v:v), vortex for 1 minute, sonicate in a 4 °C water bath for 10 minutes, centrifuge at 25,000 rpm at 4 °C for 15 minutes, and collect the supernatant in a sample vial. I n total, 50 μL of the supernatant from each sample was mixed to form an OC quality control sample, which was used to evaluate the repeatability and stability of the LC-MS analysis process.

For serum samples, add 700 μL of extraction solvent (methanol:acetonitrile: water = 4:2:1, v/v) containing internal standard 1 to 100 μL of sample. Vortex for 1 minute and store at −20 °C for 2 hours. Centrifuge at 25,000 g for 15 minutes at 4 °C. Transfer 600 μL supernatant to a new EP tube, evaporate to dryness using a vacuum evaporator, then add 180 μL methanol: pure water (1:1 v/v). Vortex for 10 minutes until completely dissolved in the reconstitution solution. Centrifuge again at 25,000 g for 15 minutes at 4 °C. centrifuge at 25,000 g for 15 minutes at 4 °C, and transfer the supernatant to a new EP tube. Finally, mix 20 μL from each sample to create an OC sample for evaluating the repeatability and stability of the LC-MS analysis process.

Metabolite detection was performed using a QExactive HF high-resolution mass spectrometer (Thermo Fisher Scientific, USA) for both primary and secondary mass spectrometry data acquisition. The full scan range was set to m/z 70–1050, with primary mass spectrometry resolution at 120,000, automatic gain control (AGC) at 3 × 10^6^, and maximum injection time at 100 ms. The top 3 ions were selected for fragmentation based on parent ion intensity to acquire MS/MS data. Secondary mass spectrometry resolution was set to 30,000, AGC to 1 × 10^5^, maximum injection time to 50 ms, and collision energy employed stepped NCE: 20, 40, 60 eV. The ion source employed electrospray ionization (ESI) mode with parameters including: sheath gas flow rate 40 arbitrary units, auxiliary gas flow rate 10 arbitrary units, spray voltage 3.80 kV (positive ion mode) and 3.20 kV (negative ion mode), ion transfer tube temperature 320 °C, auxiliary gas heating temperature 350 °C. Raw mass spectrometry data underwent peak extraction, alignment, and quantitative processing using Compound Discoverer 3.3 (Thermo Fisher Scientific). Metabolite identification was performed against the BMDB (BGI Metabolome Database), mzCloud, and ChemSpider databases, yielding a data matrix containing metabolite peak areas and identification results. The exported results were imported into metaX for data preprocessing and subsequent analysis.

### 2.4. Microbiome–metabolome correlation analysis

To ensure a one-to-one correspondence between metabolites and gut microbiota, we analyzed the omics data of subjects who underwent both metabolomics and microbiome analysis, performing a combined analysis. The association analysis was conducted at two levels: metabolite + species/microbial taxa (metabolism + metagenomics/16S-specific) and metabolite + functional genes. In these association analyzes, a comprehensive approach incorporating univariate correlations, unsupervised model-based associations, supervised model-based associations, and biological function-based associations will be employed to identify key cross-omics features associated with post-stroke depression from both statistical and biological perspectives.

### 2.5. Statistical analysis

#### 2.5.1. Metagenomic data analysis

After passing quality control, fecal samples underwent library preparation. Qualified libraries were sequenced using the DNBSEQ platform, and all sequencing data were visualized using the Dr.TOM platform. In microbiology, OTU refers to an Operational Taxonomic Unit. OTU is a method for clustering microbial sequences based on sequence similarity. Typically, a 97% sequence similarity threshold is used to define OTUs, meaning sequences with similarity above 97% are grouped into a single OTU. Alpha diversity reflects the number of species in a microbial community and the evenness of their distribution. Commonly used metrics include the Chao-1 index, Simpson's index, and Shannon index. A Kruskal–Wallis rank-sum test was used to compare the overall differences among the three groups (NPSD, Mild-PSD, and Mod-PSD); For indicators showing significant differences in the overall analysis, pairwise comparisons were further conducted using the Wilcoxon rank-sum test, with Benjamini–Hochberg correction applied for multiple comparisons. Beta diversity was calculated via PLS-DA analysis and tested using permutational multivariate analysis of variance (PMVA) with the Adonis function to demonstrate statistically significant differences in overall microbial community structure across different depressive states.

KEGG Pathway enrichment first annotated genes to the KEGG database, then identified differentially abundant KOs through significance analysis. Algorithms subsequently calculated the Reporter score—the enrichment score—for pathways containing these differential KOs. This cumulative trend reflected pathway changes, bridging micro- and macro-level insights. Wilcoxon rank-sum tests analyzed KO genes in each CKD group, comparing them against the NPSD group.

#### 2.5.2 Non-targeted metabolomics analysis

Raw metabolomics data exported from Compound Discoverer were imported into metaX for preprocessing. First, Probabilistic Quotient Normalization (PQN) was applied to correct total mass differences between samples. Subsequently, a QC-sample-based robust LOESS signal correction method (QC-RLSC) was used to correct instrument batch effects. The coefficient of variation (CV) for metabolite relative peak areas in QC samples was calculated, excluding unstable features with CV > 30%. Data underwent log2 transformation and Pareto scaling. Quality control assessment employed principal component analysis (PCA), including QC samples, to evaluate overall data distribution, intergroup separation trends, and instrument stability. QC sample clustering served as a measure of system reproducibility.

Before conducting differential analysis, to ensure the relevance and specificity of the results, we rigorously removed exogenous substances (such as dietary components) and drug metabolites from serum and fecal metabolomics data by incorporating annotation information from the HMDB and KEGG databases. Finally, we classified the metabolites by chemical and biological function using databases such as HMDB and KEGG. To explore the differences in metabolomics or microbiome characteristics among different groups, we constructed a classification model using supervised PLS-DA (based on log2 transformation and Pareto scaling) and evaluated the model's reliability using 7-fold cross-validation. Based on this, we further constructed an OPLS-DA model. Differential feature selection was based on variable projection importance (VIP) ≥ 1, the |log2FC| threshold, and the *p*-value after multiple testing correction (*P* < 0.05) to identify key metabolites or microbial biomarkers. Finally, we used KEGG Pathway enrichment analysis to identify impaired metabolic pathways and plotted ROC curves to evaluate the diagnostic efficacy of potential biomarkers.

#### 2.5.3. Correlation analysis between metabolomics and metagenomics

During the data preparation phase of the association analysis, a rigorous filtering procedure was performed on the metabolomics data to reconfirm the exclusion of exogenous and drug-related metabolites, ensuring the authenticity of inter-group correlations. Both metabolomics and metagenomics data were processed based on relative abundance before correlation analysis. Centralization and standardization were applied to reduce bias from dimensionality differences. Data preprocessing involved subtracting the mean and dividing by the standard deviation for all variables. Additionally, metabolomics data underwent log2 transformation and Pareto scaling to enhance the stability of subsequent dimensionality reduction and classification models. Inter-omics correlations were assessed using Spearman's rank correlation coefficient, which evaluates nonlinear monotonic relationships based on variable ranks and is suitable for high-dimensional, non-normally distributed omics data. Calculations were performed using the corr.test function from the R package psych, with multiple testing correction applied to the correlation matrix.

To assess data structure and sample distribution characteristics, principal component analysis (PCA) was applied to reduce dimensionality across datasets, visualizing intergroup trends and batch effects. CCA was further employed to explore linear combinations between metabolites and metagenomic functional features. The R package mixOmics identified optimal typical variables and evaluated overall synergistic variation patterns between the two omics. For feature selection, a predictive model was constructed using randomForest in R software for sampling, identifying key metabolites or microbial features distinguishing groups based on variable importance. Additionally, we evaluated the models using 10-fold cross-validation, with feature selection nested within each training fold in the joint model to minimize information leakage and optimism bias as much as possible. Model performance was quantified using AUC, sensitivity, and specificity; simultaneously, a permutation test was conducted on the rigorously validated integrated model to assess whether its discriminatory ability was significantly higher than random levels. Given the relatively limited sample size in this study and the fact that all classification models were internally validated using the same study cohort, the results of the multi-omics integration analysis should primarily be interpreted as exploratory evidence, and their robustness requires further validation in larger samples and external independent cohorts. To further analyze systematic variations in intergroup differences, PLS-DA was applied to build a supervised model, extracting latent variables most relevant to classification and evaluating their discriminative power to screen biologically meaningful differential features. Finally, KEGG pathway enrichment analysis was performed on differentially expressed metabolites and significantly correlated functional genes to identify metabolic and microbial functional pathways associated with post-stroke depression.

#### 2.5.4. Analysis of blood cytokine concentrations

Following the manufacturer's instructions (Wellgrow), a custom multiplex magnetic bead immunoassay kit (Catalog #HJC202511-6PLEX-B) was used to simultaneously quantify levels of six cytokines (IL-1β, IL-6, IL-10, IL-18, TNFα, and IFN-*γ*). Prepare an 8-point standard curve (S0–S7) using a 1:2.5 gradient dilution with Analysis Buffer I. The highest standard concentrations (S7) are set as follows: IL-1β (4300 pg/mL), IL-6 (5300 pg/mL), IL-10 (4000 pg/mL), IL-18 (5000 pg/mL), TNFα (3200 pg/mL), and IFN-*γ* (2900 pg/mL). During the assay, mix 50 μL of capture bead suspension (vortex for > 15 seconds before use) with 50 μL of standard or sample in a reaction tube. Incubate at room temperature in the dark for 1 hour. Perform magnetic separation and remove the supernatant. Add 100 μL of biotinylated antibody and incubate at room temperature in the dark for 1 hour. After washing with wash buffer, add 100 μL streptavidin-phycoerythrin (SA-PE) and incubate for 30 minutes at room temperature in the dark. Finally, resuspend the magnetic beads in 200 μL wash buffer after one wash. Perform data acquisition using a flow cytometer (BD Canto II). Analyze data with WellCKAS software and calculate cytokine concentrations based on the standard curve.

Serum CRP levels were quantified using a CRP assay kit (Registration No. 20172400484, Guangdong Pumon Biomedical Technology Co., Ltd., Shenzhen, China) via latex-enhanced immunoturbidimetry (or immunoturbidimetry). In brief, plasma samples were separated and stored at −80 °C. Testing was performed on a specific protein analyzer (Pumon PA-990 pro Specific Protein Analyzer). CRP concentration was determined by measuring changes in absorbance/turbidity, which were proportional to CRP content in the sample. Results are expressed in mg/L.

## 3. Results

### 3.1. Basic characteristics of study subjects

A total of 91 patients were enrolled in this study and categorized into a non-depressed group(NPSD) (*n* = 33), a mild depression group(*n* = 31), and a moderate depression group (*n* = 27) based on depression severity. The baseline clinical characteristics are shown in Table 1. Comparisons among the three groups regarding age, sex, stroke type, disease duration, hypertension, hyperglycemia, hyperlipidemia, and stroke lesion distribution showed no statistically significant differences (all *P* > 0.05). Baseline data across the groups were comparable.

**Table 1.** Clinical characteristics of the study population.

Variable	Non-depressed (*n* = 33)	Mild-depression (*n* = 31)	Moderate-depression (*n* = 27)	Statistic	*P*-value
Age (years)	61.5 ± 11.8	63.8 ± 11.2	63.2 ± 12.4	0.767	0.681
Male Sex, *n* (%)	20 (60.6%)	17 (54.8%)	17 (63.0%)	0.429	0.807
Ischemic Stroke, *n* (%)	16 (48.5%)	20 (64.5%)	14 (51.9%)	1.808	0.405
Hypertension	32 (97.0%)	28 (90.3%)	24 (88.9%)	1.627	0.443
Hyperglycemia	11 (33.3%)	15 (48.4%)	9 (33.3%)	1.957	0.376
Hyperlipidemia	5 (15.2%)	5 (16.1%)	6 (22.2%)	0.581	0.748
HAMD Score	4 (3.0, 6.0)	10 (8.5, 12.0)	18 (18.0, 20.0)	80.495	<0.001
SDS Score	25 (23.0, 27.0)	35 (30.0, 41.0)	50 (47.0, 56.0)	68.991	<0.001
Disease duration (days)	56.0 (22.0, 85.0)	36.0 (26.5, 76.5)	31.0 (19.5, 80.5)	0.608	0.738
Antidepressant use, *n* (%)	15 (45.5%)	22 (71.0%)	21 (77.8%)	7.776	0.020
PPI use, *n* (%)	15 (45.5%)	20 (64.5%)	23 (85.2%)	10.154	0.006
Stroke lesion distribution, *n* (%)				3.711	0.447
Subcortical	18 (54.5%)	11 (35.5%)	11 (40.7%)		
Cortical/Lobar	14 (42.4%)	16 (51.6%)	13 (48.1%)		
Multiple or Other	1 (3.0%)	4 (12.9%)	3 (11.1%)		

Consistent with the group classifications, HAMD and SDS scores increased with the severity of depression across all three groups. The median (interquartile range) HAMD scores were 4.0 (3.0, 6.0) points, 10.0 (8.5, 12.0) points, and 18.0 (18.0, 20.0) points, respectively; the median (interquartile range) SDS scores were 25.0 (23.0, 27.0), 35.0 (30.0, 41.0), and 50.0 (47.0, 56.0), respectively, supporting the validity of the severity-based grouping.

Dietary patterns and nutritional status were not systematically assessed in this study; therefore, potential residual confounding effects of dietary factors on differences in gut microbiota and metabolites cannot be ruled out. Additionally, to evaluate the potential confounding effects of medication use, this study conducted sensitivity analyzes by excluding by separately excluding participants using proton pump inhibitors (PPIs) and those using antidepressants. The results showed that some associations remained consistent across different analysis scenarios, while the statistical significance of certain inflammatory markers, metabolites, and microbiota-related features changed after excluding participants using specific medications. Specific results are presented in Supplementary Table S1.

The non-depressed, mild depression, and moderate depression groups included 33, 31, and 27 patients, respectively. Continuous variables are presented as mean ± standard deviation or median (25th percentile, 75th percentile), as appropriate, and categorical variables are presented as *n* (%). Between-group comparisons were performed using appropriate statistical methods according to variable type and distribution. HAMD, Hamilton Depression Rating Scale; SDS, Self-Rating Depression Scale; PPI, proton pump inhibitor. For stroke lesion distribution, Subcortical indicates subcortical lesions, Cortical/Lobar indicates cortical or lobar lesions, and Multiple or Other indicates multiple or other lesion locations. A two-sided *P* < 0.05 was considered statistically significant.

### 3.2. Changes in gut microbiota diversity and composition in post-stroke depression patients

#### 3.2.1. Alterations in gut microbiota diversity and overall structure

**Figure 1.**
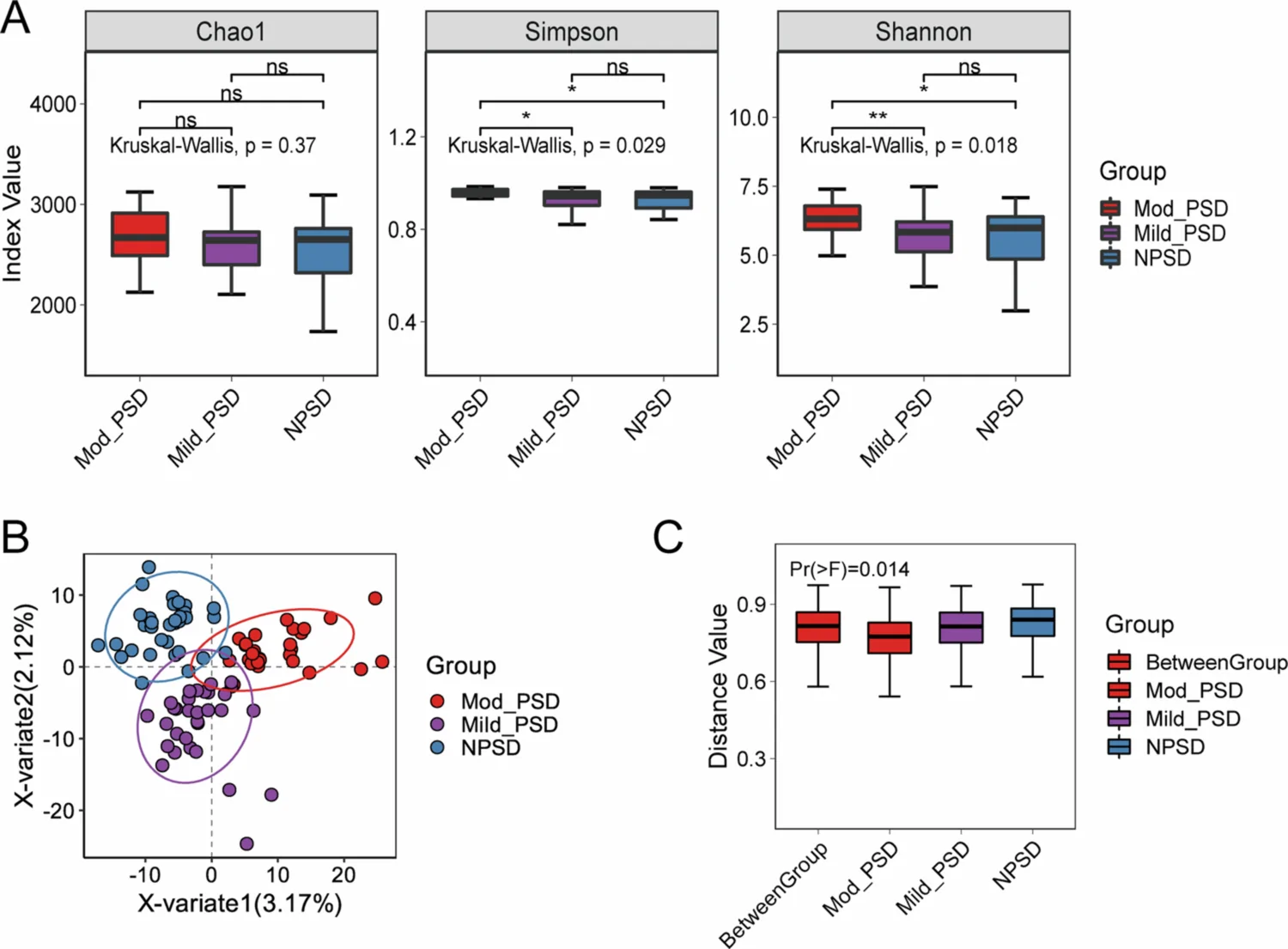
Alterations in gut microbiota diversity and community structure at the species level among patients with post-stroke depression (PSD) of varying severity. (A) Comparison of alpha diversity indices (Chao1, Simpson, and Shannon) among the moderate PSD (Mod_PSD), mild PSD (Mild_PSD), and non-PSD (NPSD) groups. (B) Partial Least Squares Discriminant Analysis (PLS-DA) score plot demonstrating distinct gut microbiota structure separation among the three groups. (C) Beta diversity analysis based on Bray-Curtis distance. Boxplots illustrate intra- and intergroup distance differences. Note: ns indicates no significant difference; *P < 0.05; **P < 0.01.

Analysis of alpha diversity at the species level revealed distinct patterns across the three groups. The Chao1 richness index showed no significant differences among the Mild-PSD, Mod-PSD, and NPSD groups (Kruskal–Wallis rank-sum test:H = 1.969, *P* = 0.37) (Figure 1A), indicating overall comparable species richness among the three groups. In contrast, Simpson and Shannon diversity indices exhibited significant intergroup differences (Simpson: *P* = 0.029; Shannon: *P* = 0.018) (Figure 1A). Further pairwise comparisons using Wilcoxon rank-sum tests followed by Benjamini–Hochberg correction revealed that the Simpson and Shannon indices in the Mod-PSD group were both significantly higher than those in the Mild-PSD and NPSD groups, whereas the differences between the Mild-PSD and NPSD groups were not statistically significant (see Supplementary Table S2A and S2B for details).

Beta diversity analysis further revealed significant heterogeneity in microbial community structure across groups. Partial Least Squares Discriminant Analysis (PLS-DA) based on Bray-Curtis distances showed clear separation among NPSD, Mild-PSD, and Mod-PSD samples (Figure 1B). The Adonis (PERMANOVA) test further confirmed significant intergroup differences (P*r* = 0.014) (Figure 1C), indicating substantial differences in overall microbial community structure across varying depression severity levels.

#### 3.2.2. Characteristic changes in the taxonomic composition of gut microbiota

**Figure 2.**
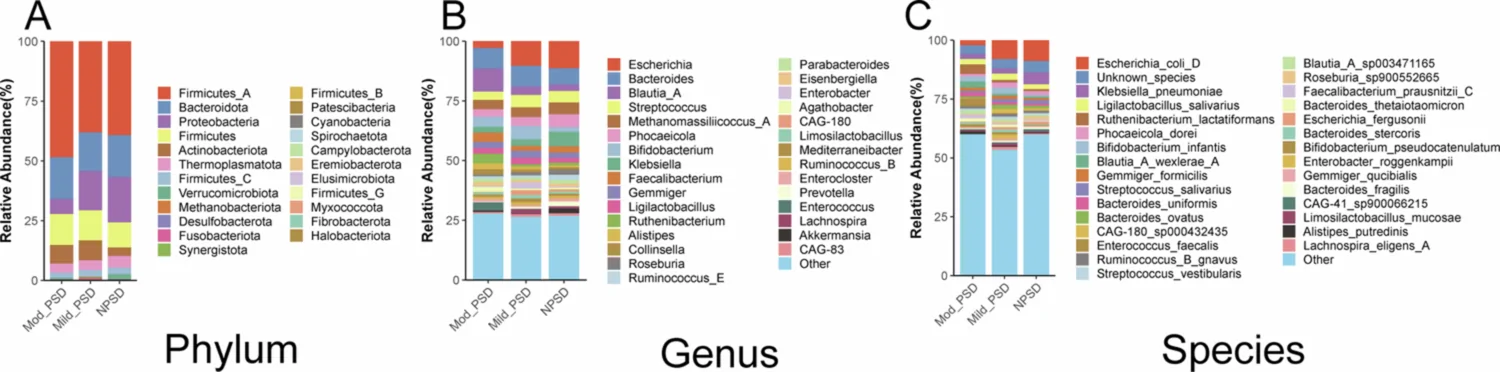
Bar charts showing relative species abundance at the Phylum, genus, and species levels for each group.

At the phylum level, all three microbial communities were dominated by Firmicutes, Bacteroidetes, and Proteobacteria (Figure 2A). The Mod-PSD group exhibited characteristic alterations: increased Firmicutes abundance alongside decreased Bacteroidetes and Proteobacteria abundance. At the genus level, the depression groups (Mild, Mod-PSD) exhibited a decreasing trend in the abundance of Escherichia, Streptococcus, and Klebsiella, with the most significant decline observed in the moderate depression group. Conversely, *Bacteroides*, Blautia_A, *Bifidobacterium*, Faecalibacterium, and *Enterococcus* were significantly enriched in the depression group (Figure 2B). At the species level, Escherichia coli, Klebsiella pneumoniae, and Ruminococcus B exhibited reduced relative abundance in the depression group, while Ruthenibacterium, *Bifidobacterium* infantis, and *Enterococcus* faecalis showed higher relative abundance in the depression group (Figure 2C).

**Figure 3.**
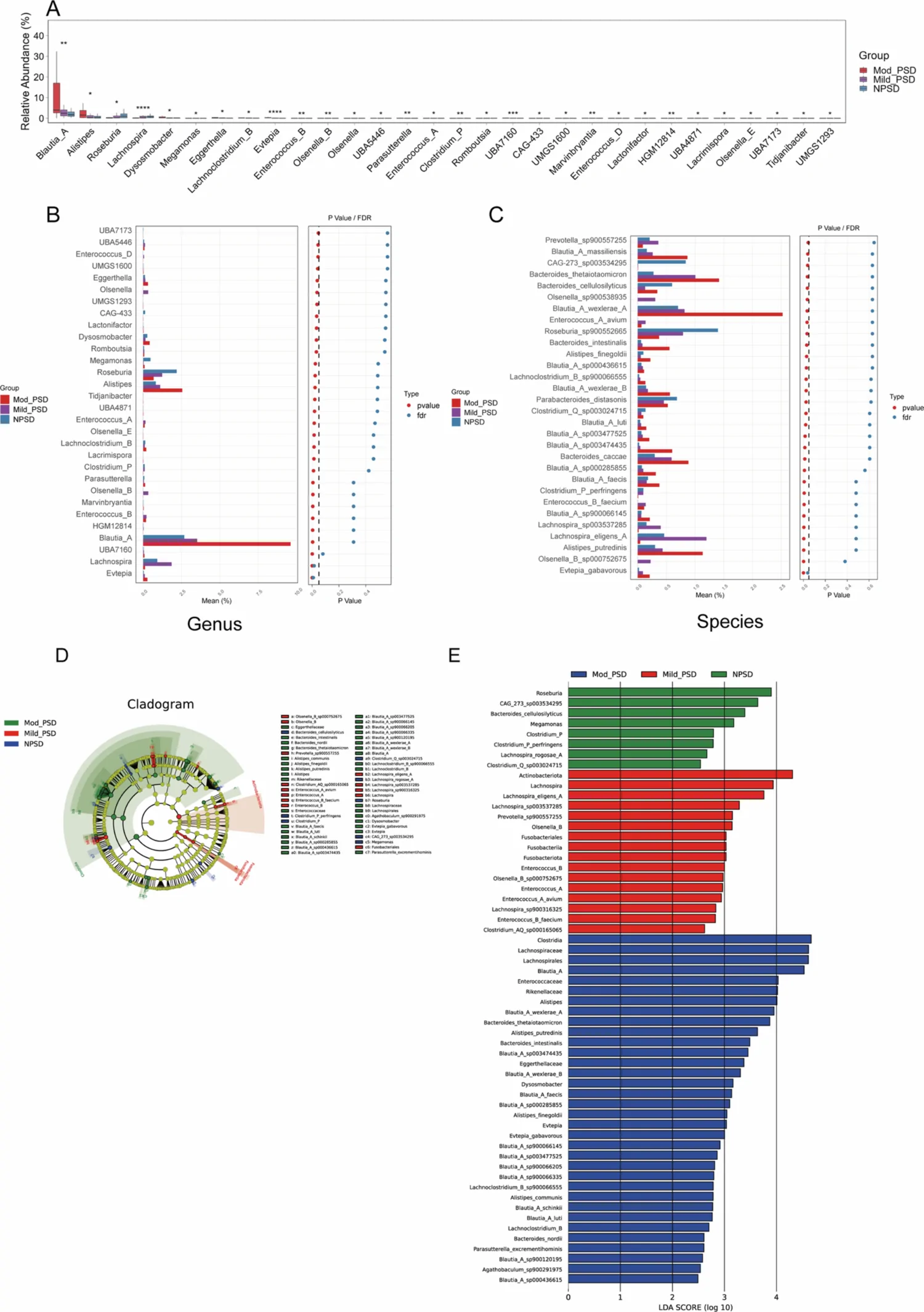
Biomarkers of the gut microbiota and their predictive functions. (A) Box plot showing significant differences in relative abundance among Prostaglanda genera. (B, C) STAMP analysis showing significant differences in species at the genus level (B) and species level (C) (FDR < 0.05). (D) Cladogram of the gut microbiota. Concentric circles radiating outward from the innermost circle represent taxonomic levels such as phylum, class, order, family, genus, and species. Each node represents the species classification at that level, and the size of the node represents the species richness. Colored nodes indicate significant enrichment in the Mod_PSD group (green), Mild_PSD group (red), or NPSD group (blue) (LEfSe analysis: *P* < 0.05). (E) Bar chart of LDA value distribution. The vertical axis represents the taxonomic units with differences, and the horizontal axis represents the LDA value; a larger value indicates a greater difference between groups.

In the species-level differential analysis, the box plots revealed the most pronounced changes in the genus levels among the three groups for Blautia_A and *Alistipes* (Figure 3A). Notably, the STAMP analysis revealed the most significant differences at the genus level among the three groups for *Lachnospira* and Evtepia (FDR < 0.05, Figure 3B). At the species level, the difference in Evtepia_gabavorous (which feeds only on GABA bacteria, also known as Firmicutes bacterium CAG:114) was statistically significant (FDR < 0.05, Figure 3C). Linear discriminant analysis Effect Size (LEfSe) identified the species most explanatory of group differences (termed biomarkers) across Mild-PS, Mod-PSD, and NPSD groups, along with their relative contribution to group variation (LDA; Figure 3D). Potential biomarkers with LDA values > 3 are depicted in Figure 3E.

#### 3.2.3. Differential analysis of gut microbiota functional genes and metabolic pathways

**Figure 4.**
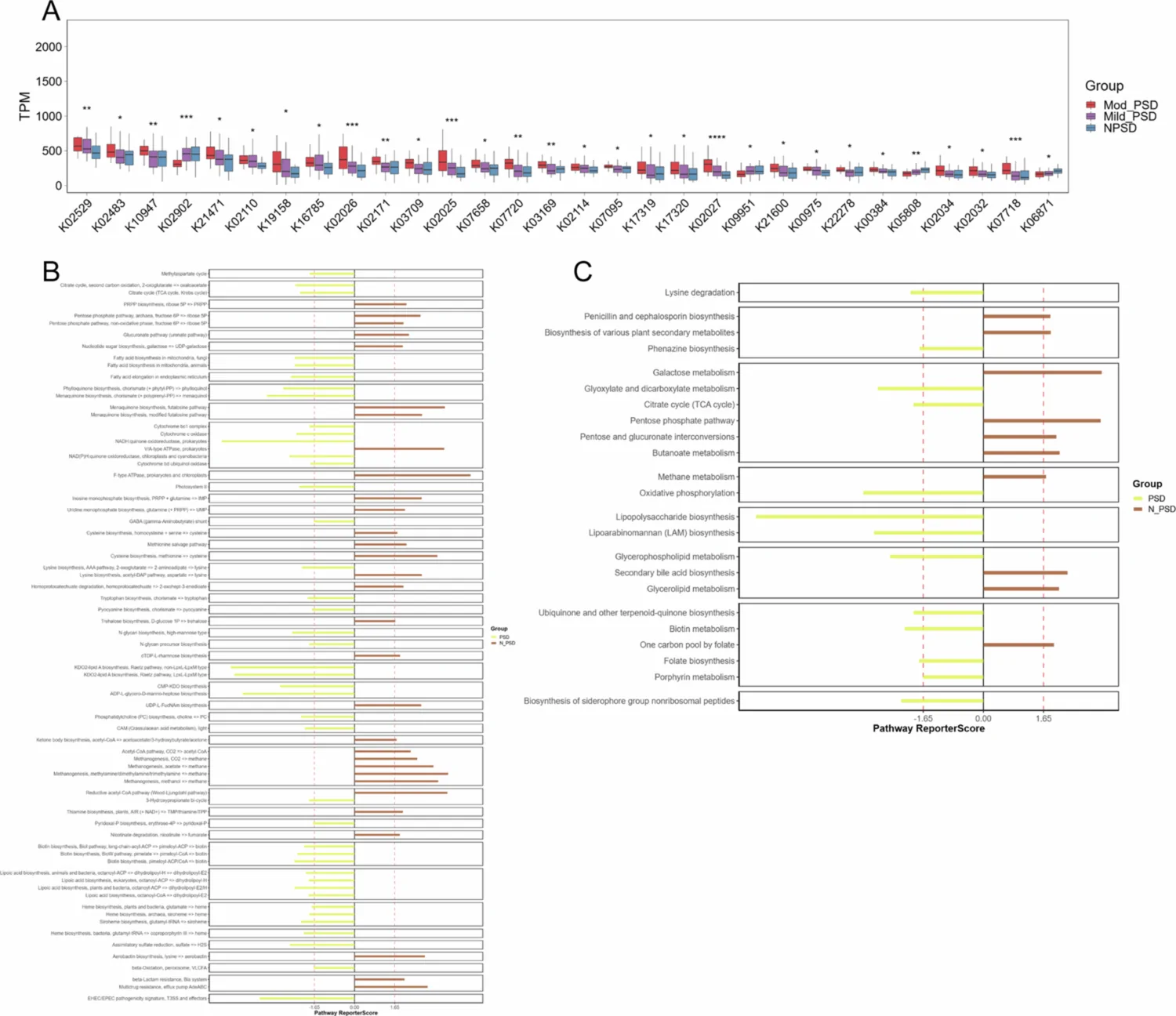
Functional prediction and metabolic pathway analysis of gut microbiota. (A) Functional difference box plots. The x-axis represents statistically significant functional categories, while the y-axis shows relative abundance. Stars above bars indicate functional differences in abundance between the corresponding sample and other groups. Star symbols denote significance levels: **** indicates *P* < 0.0001, *** indicates *P* < 0.001, ** indicates *P* < 0.01, * indicates *P* < 0.05, ns indicates *P* > 0.05, NS indicates *P* = 1. (B, C) Functional modules and metabolic analysis based on KEGG Reporter Score. The x-axis represents Reporter Score values, and the y-axis represents metabolic pathways. Vertical dashed lines indicate the significance threshold (typically 1.65). Note: In (B) and (C), yellow bars (negative values) indicate enrichment in the PSD group, while brown bars (positive values) indicate enrichment in the NPSD group. Only functional categories exceeding the Reporter Score threshold are displayed.

Analysis of functional gene (KO) levels revealed significant gradients in the abundance of several key functional genes among the three groups (*P* < 0.05). The Mod-PSD group showed widespread upregulation of functional abundance, particularly in KOs related to basal metabolism, transmembrane transport, and stress response (e.g., K02529, K02483, K10947, K21411, K16785, K02026, K02025, K03169, K17320), with significantly higher abundance than the Mild-PSD and NPSD groups (Figure 4A). Conversely, some functional genes involved in host homeostasis maintenance (e.g., K02032, K06871) were significantly suppressed in the PSD group.

Reporter Score analysis based on the KEGG database revealed significant functional remodeling of the gut microbiota in PSD patients. Functional enrichment results showed that the most significant alterations in the PSD group were concentrated in lipopolysaccharide (LPS) synthesis-related modules. Furthermore, the PSD group also exhibited differences in energy metabolism and fermentation-related pathways, such as butyrate metabolism (map00650, Score = 2.09); simultaneously, differentially expressed carbon metabolism pathways such as the pentose phosphate pathway (map00030, Score = 3.22) and pentose-glucuronide tautomerism (map00040, Score = 2.00) were also detected (Figure 4B and C).

### 3.3. Clinical correlations of serum inflammatory markers and multi-omics interaction characteristics

We first assessed the relationship between systemic inflammation levels and PSD severity. In a cross-sectional comparison of the NPSD, Mild-PSD, and Mod-PSD groups, no statistically significant differences in inflammatory markers were observed between subgroups (Kruskal–Wallis rank-sum test, *P* > 0.05). Spearman's correlation analysis was then performed to assess associations between clinical scale scores and inflammatory markers, followed by Benjamini–Hochberg FDR correction. After FDR correction, neither depression severity group nor HAMD and SDS scores showed significant correlations with CRP, IL-18, TNF-*α*, IL-6, IL-10, IFN-*γ*, or IL-1β (Table 2).

**Table 2.** Correlation between inflammatory factors and clinical symptoms.

Variable	CRP	IL-18	TNF-*α*	IL-6	IL-10	IFN-*γ*	IL-1β
GROUP	0.089	0.127	0.033	0.212	0.228	0.137	0.201
HAMD	0.037	0.118	0.076	0.222	0.182	0.119	0.189
SDS	0.029	0.102	0.013	0.213	0.139	0.055	0.109

Note: Values are Spearman correlation coefficients. *P* values were adjusted for multiple comparisons using the Benjamini–Hochberg false discovery rate method. No correlation remained statistically significant after FDR correction. Abbreviations: HAMD, Hamilton Depression Rating Scale; SDS, Self-Rating Depression Scale; CRP, C-reactive protein.

**Figure 5.**
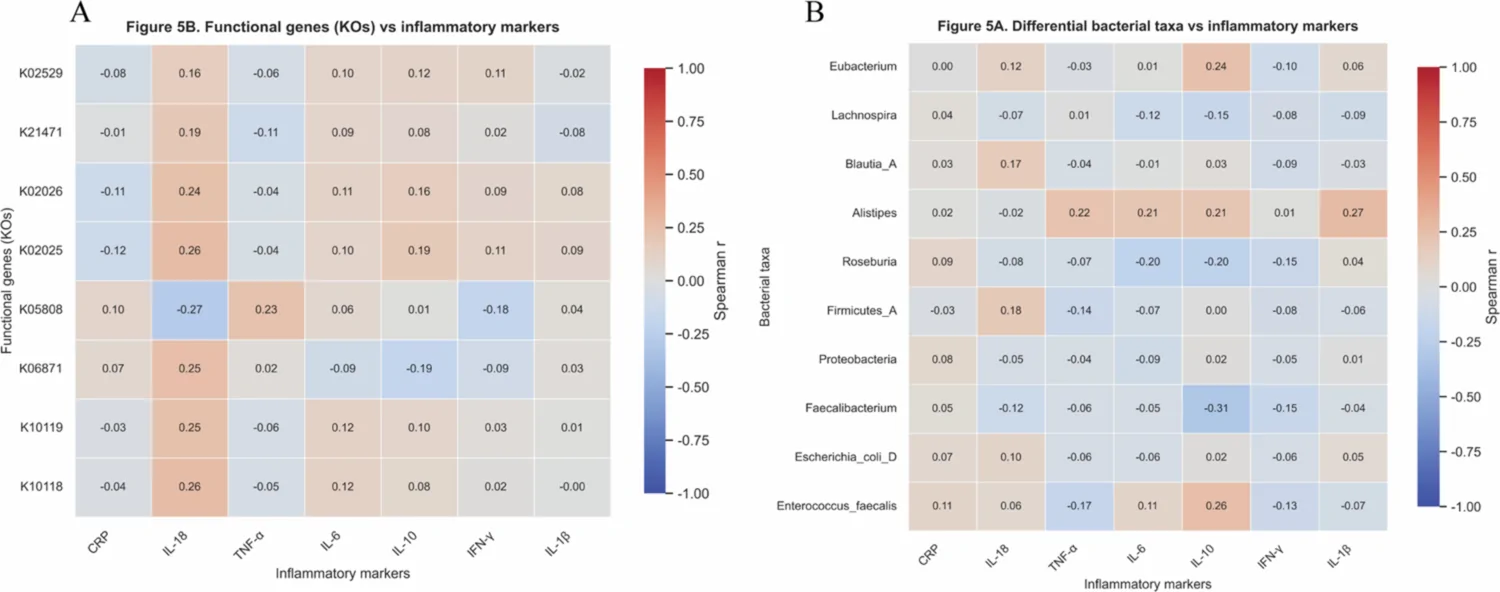
(A). Spearman correlation heatmap between gut microbiota and serum inflammatory factors. The x-axis represents inflammatory factors, and the y-axis represents different bacterial genera or species. (B). Spearman correlation heatmap between functional genes (KO) and serum inflammatory factors. The x-axis represents inflammatory factors, and the y-axis represents functional genes (KEGG Orthology, KO). The colors in the heatmap represent the Spearman correlation coefficient (r): Red indicates a positive correlation, blue indicates a negative correlation, and darker shades indicate a stronger correlation. All *P*-values were FDR-corrected using the Benjamini–Hochberg method. Since none of the correlations reached statistical significance after FDR correction, significance markers are not shown in the figures. Results with stronger correlations (∣r∣ ≥ 0.20) are summarized in Supplementary Table S3A and S3B.

To further assess the correlations between differentially abundant microbial taxa, functional genes, and inflammatory factors, Spearman's correlation analysis with Benjamini–Hochberg FDR correction was used to analyze the correlations among differentially abundant microbial taxa, functional genes (KO), and serum inflammatory factors (CRP, IL-18, TNF-*α*, IL-6, IL-10, IFN-*γ*, and IL-1β). The results showed that, in the unadjusted analysis, some microbial communities and functional genes exhibited trends of correlation with inflammatory factors, including a weak positive correlation between *Alistipes* and TNF-*α*, IL-6, IL-10, and IL-1β; a positive correlation between Eubacterium and IL-10; a negative correlation between *Faecalibacterium* and IL-10; and positive correlations of K02025, K02026, K06871, K10118, and K10119 with IL-18, and K05808 showing a negative correlation with IL-18. However, after correction for multiple comparisons, none of these correlations reached statistical significance (all FDR-adjusted *P* > 0.05). In addition, no significant correlations were observed between CRP and differentially abundant microbial taxa or functional genes (Figure 5A,B; Supplementary Table S3A and S3B).

### 3.4. Non-targeted metabolomics characteristics

#### 3.4.1. Overall analysis and differential screening of fecal and serum metabolism

**Figure 6.**
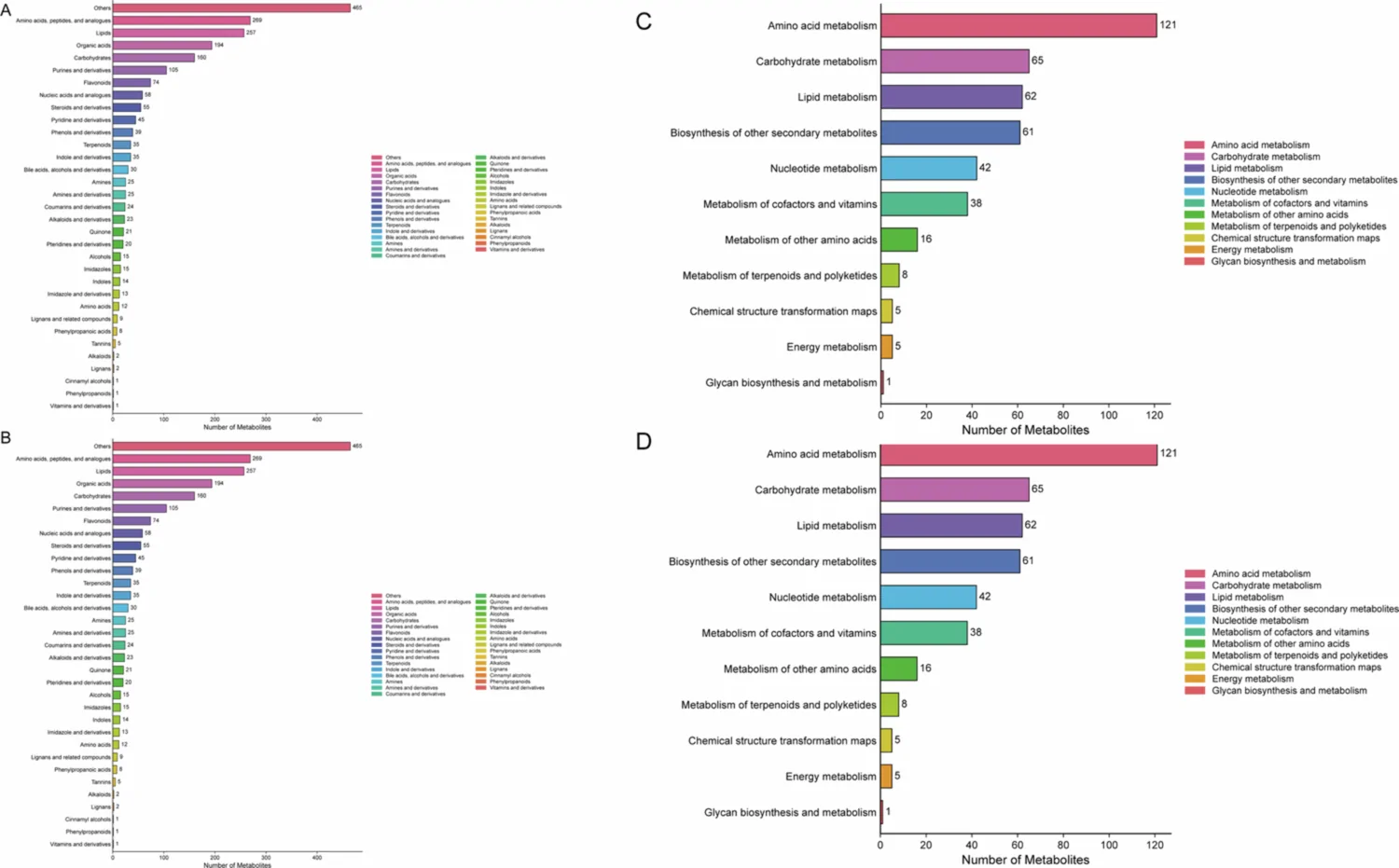
Bar charts showing metabolite classification and function. (A, B) Bar charts of metabolite chemical classification based on the Human Metabolome Database (HMDB), where the vertical axis represents metabolite class and the horizontal axis represents metabolite count. (C, D) Bar charts of metabolite functional classification based on KEGG pathways. The vertical axis represents the category of metabolic pathways, while the horizontal axis shows the number of metabolites.

Fecal and serum metabolomes demonstrated remarkable compositional alignment. Major metabolite categories in both sample types included amino acids, peptides, lipids, organic acids, and carbohydrates (Figure 6A,B). Functional analysis revealed convergent enrichment in amino acid, carbohydrate, and lipid metabolism (Figure 6C,D), reflecting the metabolic integration between the gut and the circulatory system.

#### 3.4.2. Screening of differentially metabolized compounds across groups and model validation

**Figure 7.**
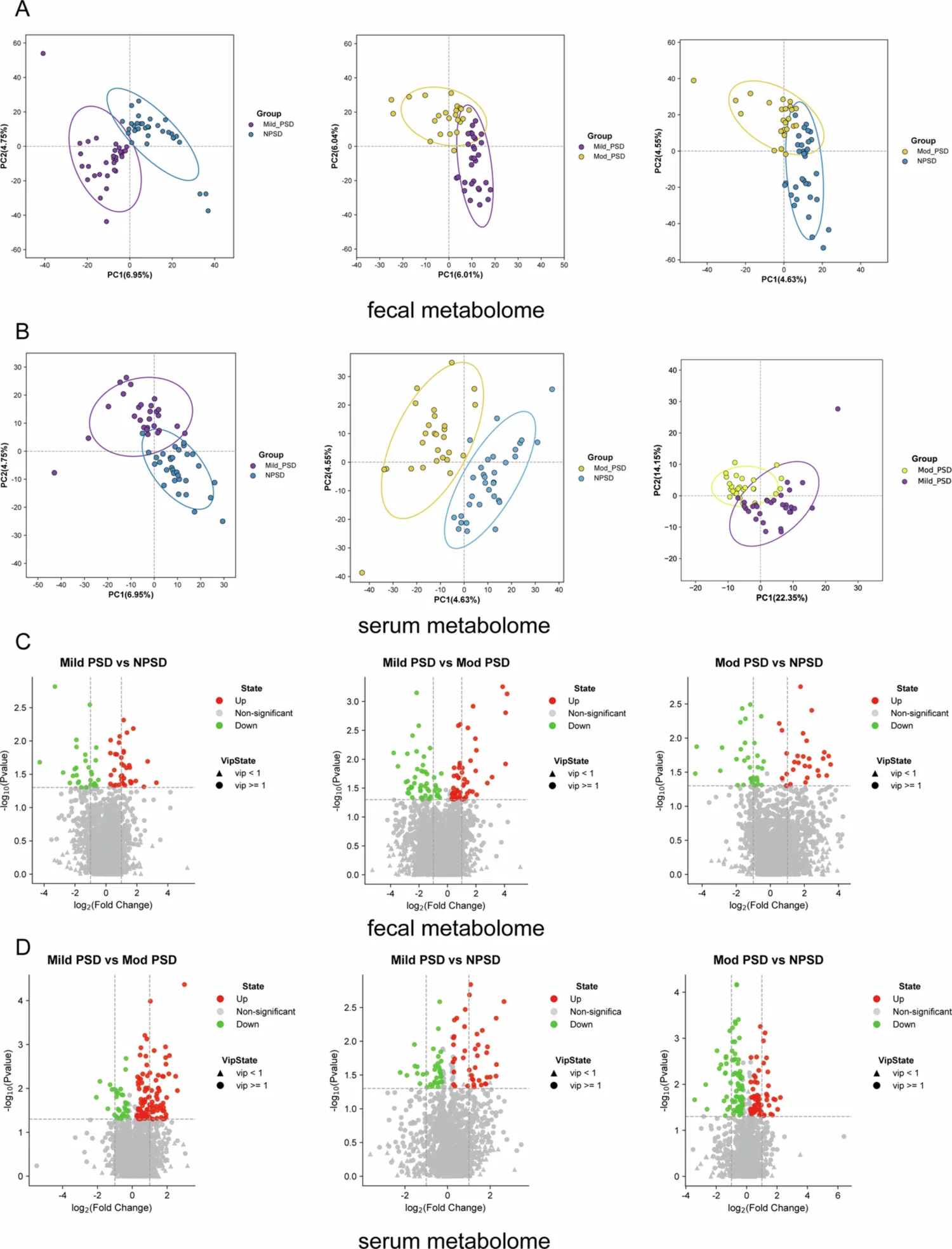
Multivariate and univariate statistical analyses of fecal and serum metabolomes. (A, B) Partial least squares discriminant analysis (PLS-DA) score plots for fecal (A) and serum (B) samples. The plots demonstrate distinct metabolic separation trends between pairwise comparisons of Mild_PSD (purple), Mod_PSD (yellow), and NPSD (blue) groups. (C, D) Volcano plots of differentially abundant metabolites in fecal (C) and serum (D) samples. The x-axis represents log2-transformed fold change (FC), while the y-axis displays -log10-transformed *p*-values. Pink dots indicate significantly upregulated metabolites, blue dots denote significantly downregulated metabolites, and gray dots represent metabolites without significant differences. Circles denote metabolites with variable importance in projection (VIP) ≥ 1, while triangles indicate metabolites with VIP < 1. The screening criteria for differential metabolites were set as: VIP ≥ 1, *P* < 0.05, and FC ≥ 1.2 or ≤ 0.83.

PLS-DA analysis revealed distinct clustering and clear separation among the NPSD, Mild-PSD, and Mod-PSD groups in both fecal and serum metabolomes (Figure 7A,B).

Next, univariate differential analysis was performed, with screening criteria set as VIP ≥ 1, Fold Change (FC) ≥ 1.2 or ≤0.83, and *P*-value < 0.05. In feces, the most significant variation occurred between the Mild- and Mod-PSD groups (103 metabolites), exceeding the differences found in comparisons with the NPSD group (Figure 7C). A similar trend was observed in serum, where the Mild-PSD group exhibited 135 differential metabolites compared to the Mod-PSD group (102 upregulated, 33 downregulated), highlighting a progressive metabolic shift correlating with disease severity (Figure 7D).

#### 3.4.3. Dynamic metabolic characteristics and diagnostic value of PSD severity

This study integrated Z-score normalization, KEGG enrichment, DA Score, and network topology analyzes to elucidate the dynamic evolution of the gut-peripheral blood metabolic axis across varying severities of PSD.

**Figure 8.**
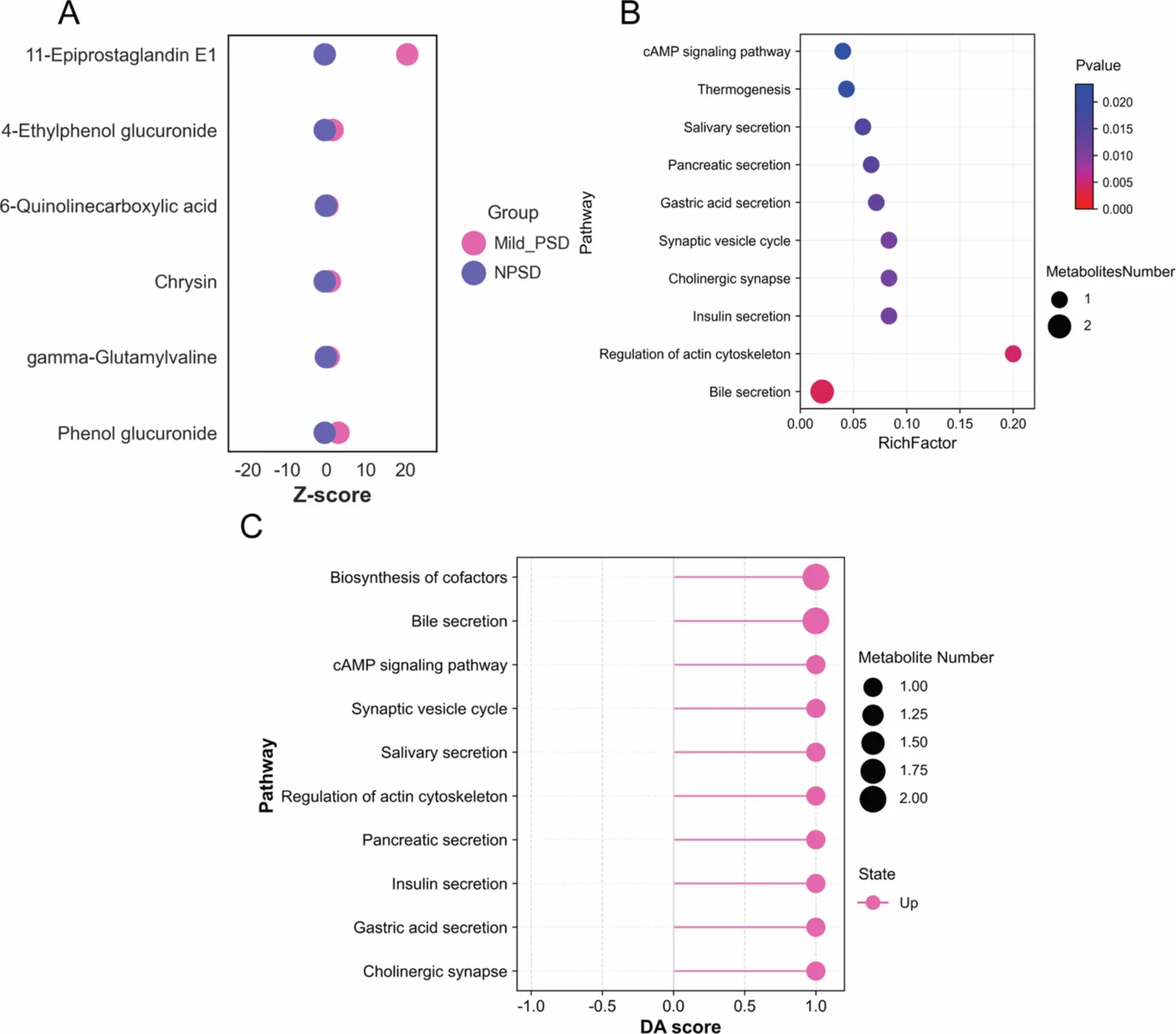
Fecal metabolic characteristics in the Mild-PSD group. (A) Content **analysis Z-score plot**, used to measure the relative abundance of metabolites at the same level. Each point represents a sample, with colors denoting different groups. The x-axis represents standardized relative abundance (Z-score). (B) KEGG pathway enrichment analysis bubble plot of fecal differential metabolites. Bubble size represents metabolite count; color gradient indicates significance level (*P*-value). (C) Enrichment analysis pathway score plot for differential abundance score analysis of the top 10 significantly enriched metabolic pathways across comparison groups (using all data if fewer than 10 pathways). The Y-axis shows metabolic pathway names, while the X-axis displays differential abundance scores (DAscore). Line length represents the absolute value of the DA score, and dot size indicates the number of metabolites. A score of +1 indicates an overall upregulation trend for all annotated differentially expressed metabolites in the pathway, while a score of −1 indicates a downregulation trend.

In the mild PSD group, fecal metabolites were significantly enriched in neuroactive compounds. Z-score analysis identified elevated levels of 11-epiprostaglandin E1 and phenol glucuronide, alongside reduced gamma-glutamylvaline (Figure 8A). KEGG pathway analysis revealed that differential metabolites were primarily involved in cholinergic synapses, the synaptic vesicle cycle, bile secretion, gastric acid secretion, and insulin secretion. Notably, neural signal transduction and digestive secretion emerged as the two dominant metabolic themes, both exhibiting an overall upregulated state (DA score = 1.0; Figure 8B,C). Furthermore, to further illustrate the potential association patterns between key differentially expressed metabolites, enzymes, and enriched pathways, this study conducted a network topology analysis; the relevant results are shown in Supplementary Figure S1A. Network analysis is primarily used to assist in identifying potential key nodes and their interconnections, providing clues for subsequent mechanistic studies; however, it does not constitute causal evidence.

**Figure 9.**
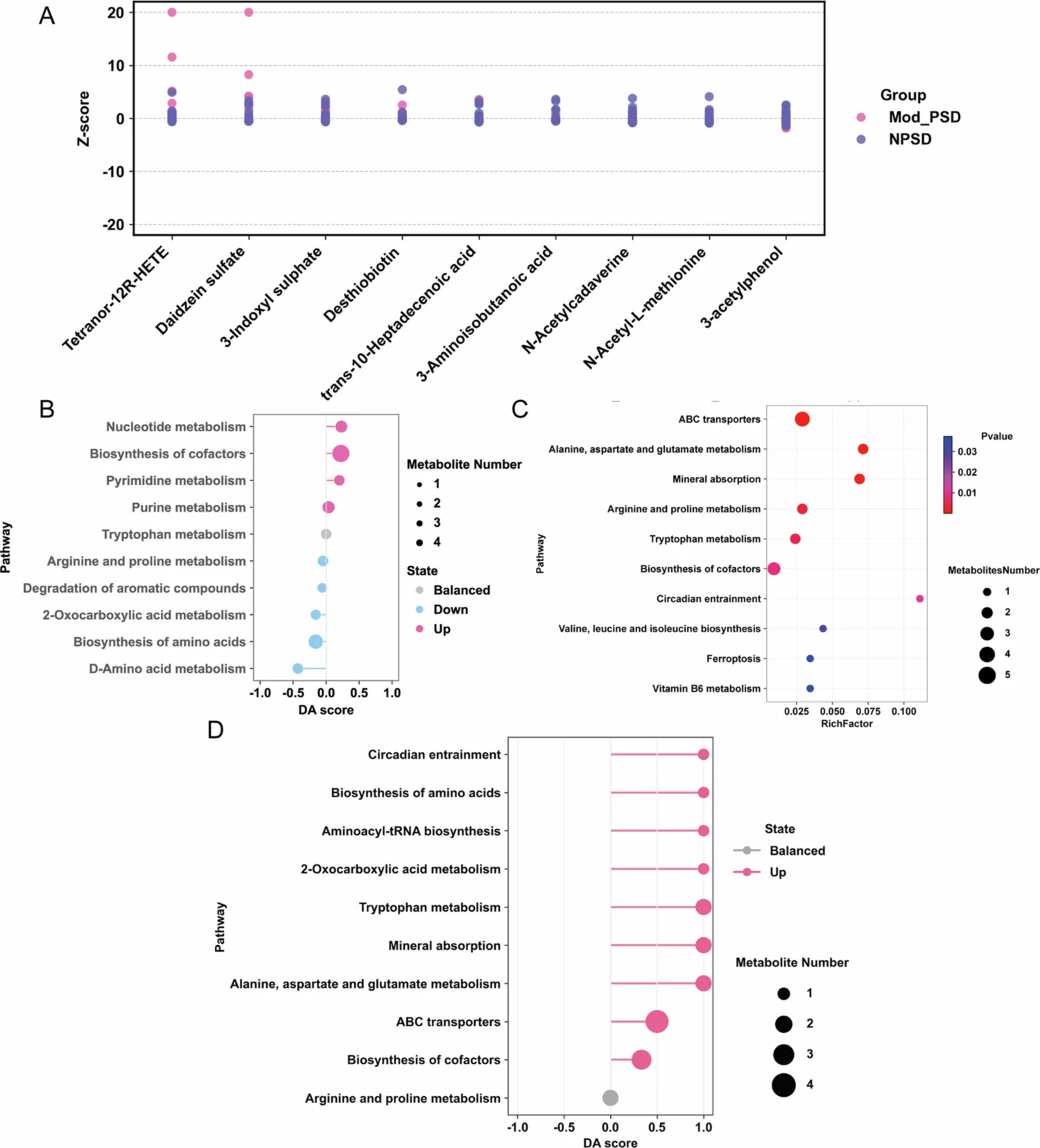
Fecal metabolic characteristics of the Mod-PSD group compared with the Mild-PSD and NPSD groups. (A) Content **analysis Z-score plot**, used to measure the relative abundance of metabolites at the same level. Each point represents a sample, and the color indicates different groups. The horizontal axis represents the standardized relative abundance (Z-score). (B, D) Enrichment analysis - pathway score plot. The top 10 significantly enriched metabolic pathways in each comparison group (if fewer than 10, all data are used) are analyzed for differential abundance scores. In the figure, the Y-axis represents the name of the metabolic pathway, and the X-axis represents the differential abundance score (DAscore). The DAscore is the overall change of all metabolites in a certain metabolic pathway. The length of the line segment represents the absolute value of the DA score, and the size of the dot represents the number of metabolites. A score of 1 indicates that the expression trend of all annotated differential metabolites in the pathway is upregulated, and a score of −1 indicates that the expression trend of all annotated differential metabolites in the pathway is downregulated. (C) Enrichment analysis - bubble chart. The X-axis enrichment factor is calculated by dividing the number of differentially expressed metabolites annotated to that pathway by the total number of metabolites in that pathway. A higher value indicates a greater proportion of differentially expressed metabolites annotated to that pathway. The size of the dots represents the number of differentially expressed metabolites annotated to that pathway.

In the moderate depression group, intestinal metabolic dysregulation was markedly exacerbated. Specifically, tetranor-12R-HETE and daidzein sulfate accumulated significantly, whereas 3-indoxyl sulfate, 3-aminoisobutanoic acid, and N-acetyl-L-methionine were depleted (Figure 9A). Pathway analysis indicated an upregulation of biotin and cofactor biosynthesis, contrasting with a downregulation trend in amino acid biosynthesis (Figure 9B). Direct comparison between Mild- and Mod-PSD groups highlighted significant enrichment in ABC transporters and alanine, aspartate, and glutamate metabolism (Figure 9C). Moreover, tryptophan metabolism, ABC transporters, and mineral absorption were all significantly upregulated (Figure 9D). Furthermore, the results of the topological analysis of the metabolite–enzyme–pathway network associated with significantly altered metabolites are shown in Supplementary Figure S1B. Within the network, N-acetylaspartic acid, isovalerylcarnitine, and D-aspartic acid formed the major hubs of the network.

**Figure 10.**
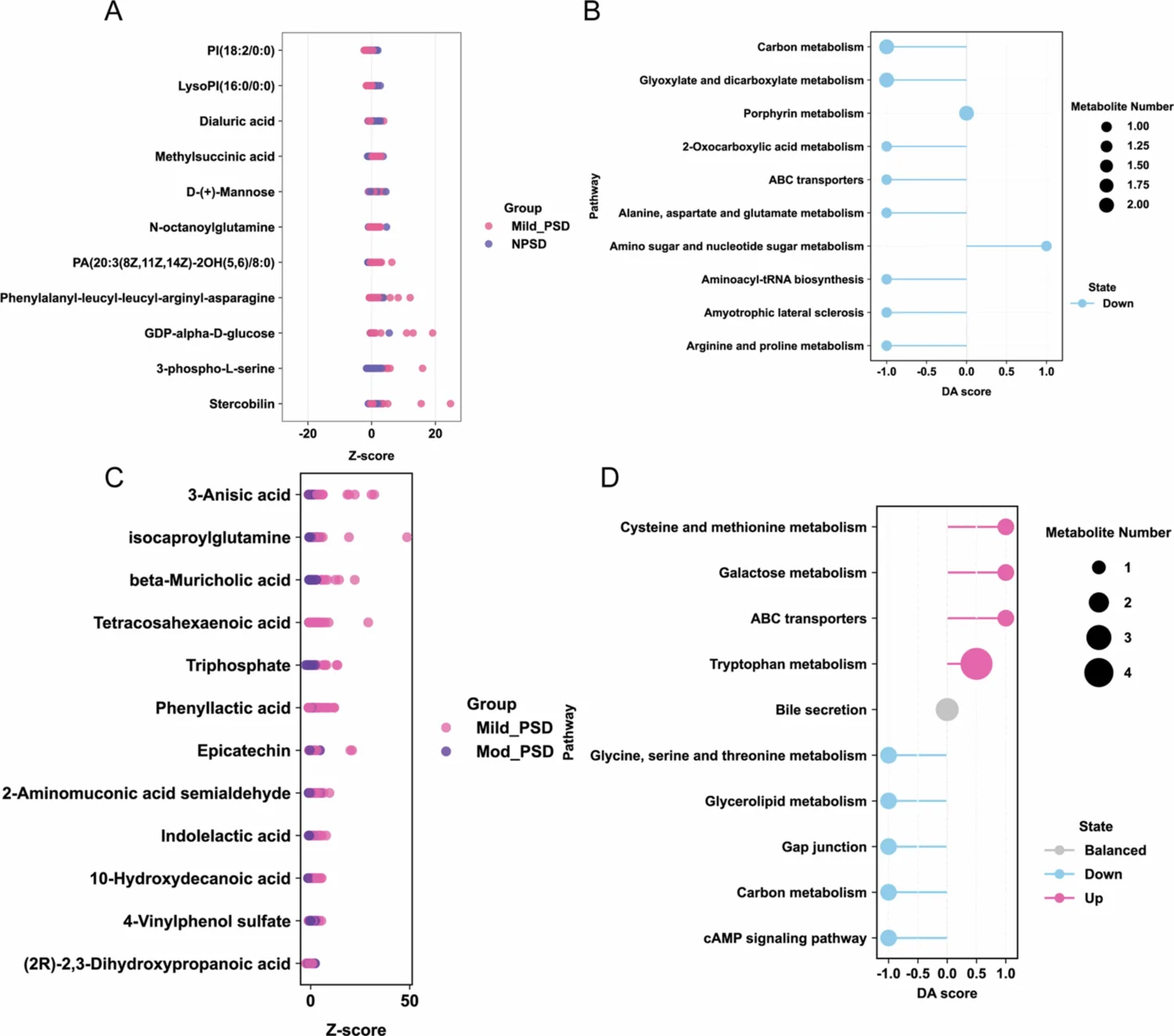
Comparison of serum metabolic characteristics among different degrees of depression. (A, C) Content analysis Z-score plot, used to measure the relative abundance of metabolites at the same level. Each point represents a sample, and the color indicates different groups. The horizontal axis represents the standardized relative abundance (Z-score). (B, D) Enrichment analysis pathway score plot. Differential abundance score analysis was performed on the top 10 significantly enriched metabolic pathways in each comparison group (if fewer than 10, all data were used). In the figure, the Y-axis represents the name of the metabolic pathway, and the X-axis represents the differential abundance score (DAscore). The length of the line segment represents the absolute value of the DA score, and the size of the dot represents the number of metabolites. A score of 1 indicates that the expression trend of all annotated differential metabolites in the pathway is upregulated, and a score of -1 indicates that the expression trend of all annotated differential metabolites in the pathway is downregulated.

In contrast to the active shifts in intestinal metabolism, serum metabolism in the mild PSD group may be characterized primarily by alterations in lipid metabolites. Specifically, we observed increased abundance of phospholipids, including PI(18:2/0:0) and LysoPI(16:0/0:0), alongside stercobilin (Figure 10A). Functional analysis revealed significant enrichment but overall downregulation (DA score < 0) of core energy pathways, such as glyoxylate and dicarboxylate metabolism and carbon metabolism (Figure 10B). Network topology analysis identified 4-ethoxybenzaldehyde and glycolic acid as central nodes (Figure 10C). In contrast to the active shifts in intestinal metabolism, the Mod-PSD groups serum metabolism was characterized primarily by alterations in lipid profiles. Specifically, we observed increased abundance of phospholipids, including PI(18:2/0:0) and LysoPI(16:0/0:0), alongside stercobilin (Figure 10A). Functional analysis revealed significant enrichment but overall downregulation (DA score < 0) of core energy pathways, such as glyoxylate and dicarboxylate metabolism and carbon metabolism (Figure 10B). In the moderate group (Mod-PSD), Z-score cluster analysis revealed a further decrease in indolelactic acid levels (Figure 10C), while the tryptophan metabolism pathway was enriched (DA score > 0, Figure 10D). Topological analysis of the network associated with key differential metabolites are shown in Supplementary Figure S1C.

Based on the above results, tryptophan metabolism and ABC transporters were identified as key pathways commonly enriched in both feces and serum (*P* < 0.05), suggesting a potential role for these pathways in linking gut microbiota alterations with host metabolic profiles. A systematic screening of tryptophan-related metabolites was conducted conducted a systematic screening of tryptophan-related metabolites in the untargeted serum and fecal metabolomic profiles of serum and feces. Detailed results at the metabolite level are presented in Supplementary Tables S4 and S5, respectively, and evidence across tryptophan metabolic branches is summarized in Supplementary Table S6. Based on the above metabolic profiles, representative metabolites were further selected for supplementary ROC curve analysis; the results suggest that some serum metabolites may have exploratory value in discriminating between different depression severities. Specific results are shown in Supplementary Figure S2.

### 3.5. Correlation between gut microbiota and metabolites

#### 3.5.1. Association analysis of gut microorganisms and fecal metabolites

**Figure 11.**
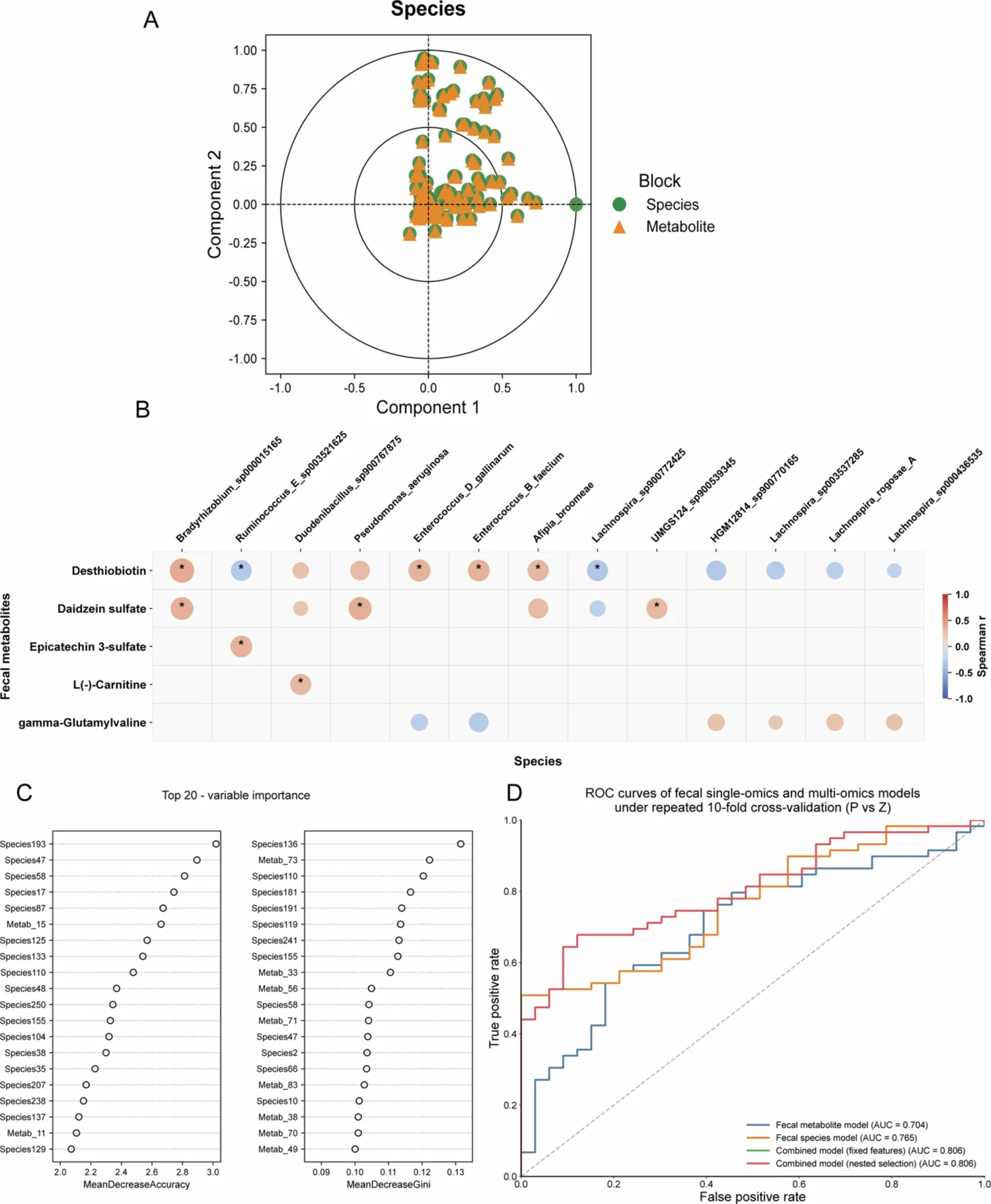
Multi-omics correlation analysis at the species level between gut microbiota and fecal metabolites. (A) Canonical Correlation Analysis (CCA) plot. Green dots represent metagenomic species; orange triangles represent metabolites. The inner circle represents a correlation coefficient of 0.5, while the outer circle represents a correlation coefficient of 1. With the dot as the center, a line segment connecting two points forms an angle. (B) Spearman correlation heatmap between differentially abundant species (rows) and metabolites (columns). Colors transition from blue to red, indicating correlation coefficients ranging from negative to positive. Significance levels are marked as *P < 0.05 and **P < 0.01. (C) Variable importance analysis based on a random forest classifier. Left panel: Mean Decrease in Accuracy Right panel: Mean Decrease in Gini. Higher values indicate greater importance of the variable in distinguishing PSD states. (D) The ROC curves based on the random forest model were internally validated using 10-fold cross-validation. The figure shows the discriminatory performance of the fecal metabolite model, the fecal species model, the combined fixed-feature model, and the combined nested-feature screening model, with AUC values of 0.704, 0.765, 0.806, and 0.806, respectively.

Canonical correlation analysis (CCA) revealed significant covariation between the overall structures of metagenomic species and fecal metabolites (Figure 11A), suggesting that gut microbiota composition is closely linked to metabolic phenotype. Spearman's correlation analysis was further employed to assess pairwise associations between differentially abundant species and differential fecal metabolites, followed by Benjamini–Hochberg correction for multiple comparisons. Considering that some metabolites may originate from drugs, chemical reagents, or other potential exogenous exposures, explicitly identified exogenous metabolites were excluded from key result summaries and visualizations to enhance the biological interpretability of the results. The revised results showed that multiple species–metabolite correlations remained statistically significant after multiple comparison correction (Figure 11B; Supplementary Table S7).

Leveraging these associations, we constructed a random forest classification model to evaluate classification performance. Variable importance analysis highlighted Species193 (CAG-312 sp002437405) as the top contributor to MeanDecreaseAccuracy, while Species136 (*Paraeggerthella hongkongensis*) led in MeanDecreaseGini, marking them as critical discriminators of PSD states (Figure 11C). Under 10-fold cross-validation (Figure 11D), the AUCs of the fecal metabolite model and the fecal species model were 0.704 and 0.765, respectively, while the combined model achieved an AUC of 0.806 under both the fixed-feature and nested-feature selection strategies. Further permutation tests showed that the combined nested model had an empirical *P*-value of 0.004975 and a 95% CI of 0.714–0.880, suggesting that its overall performance was significantly higher than the level of random classification. Compared with single-omics models, the combined model showed higher discriminatory power.

**Figure 12.**
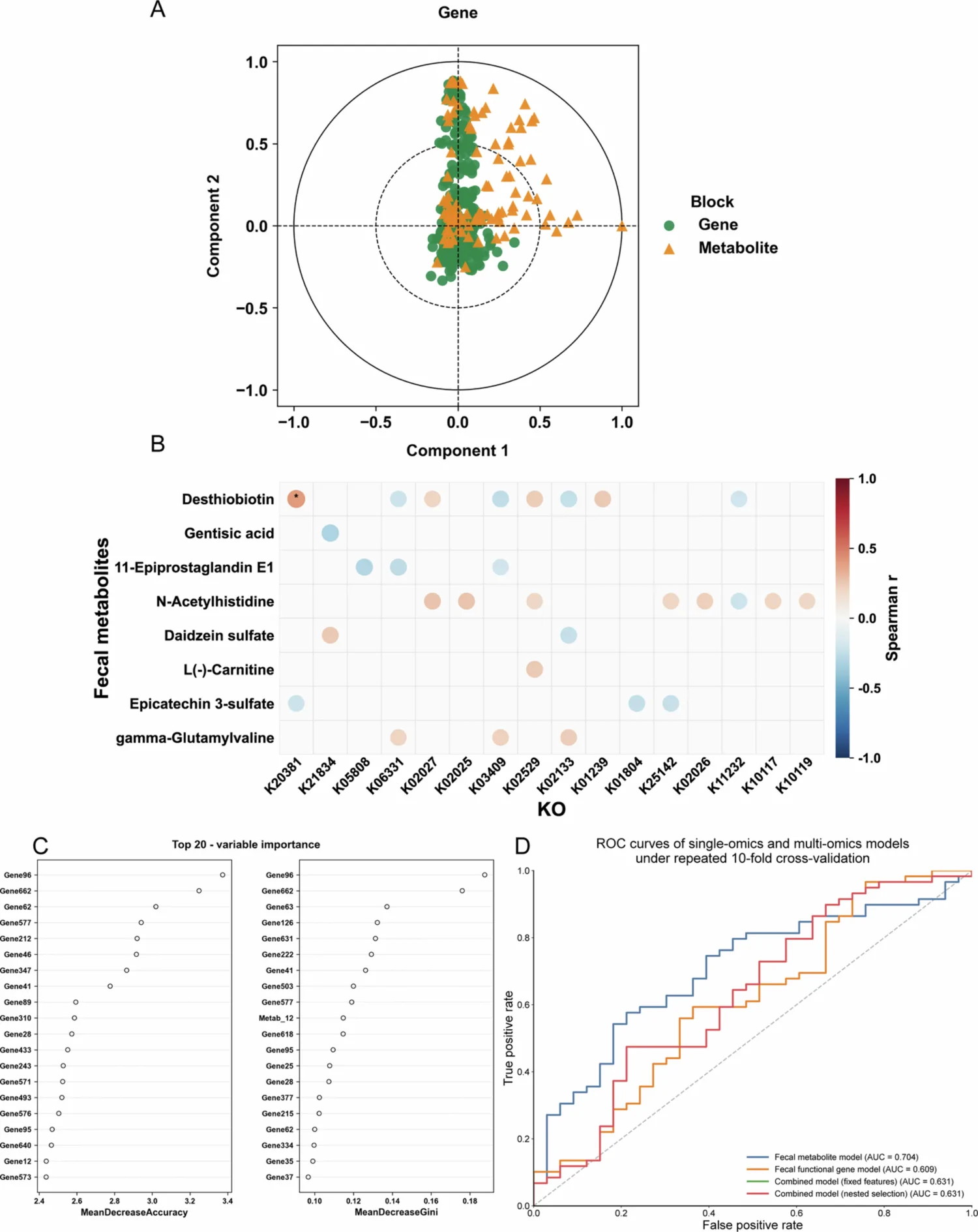
Multi-omics correlation analysis of gut microbiota and fecal metabolites at the functional gene level. (A) Canonical Correlation Analysis (CCA) plot. Green dots represent metagenomic functional genes, orange triangles represent metabolites. The inner circle represents a correlation coefficient of 0.5, while the outer circle represents a correlation coefficient of 1. (B) Spearman correlation heatmap between **differential functional genes** (rows) and metabolites (columns). Colors transition from blue to red, indicating correlation coefficients ranging from negative to positive. Significance levels are marked as *P < 0.05 and **P < 0.01. (C) Variable importance analysis based on a random forest classifier. Left panel: Mean Decrease in Accuracy (MDAcc). Right panel: Mean Decrease in Gini (MDGini). Higher values indicate greater importance of the variable in distinguishing PSD states. (D) ROC curves for the random forest model, with internal validation performed using 10-fold cross-validation. The AUC values for the fecal metabolomics model, the fecal functional gene model, the fixed-feature combined model, and the nested-feature screening combined model were 0.704, 0.609, 0.631, and 0.631, respectively.

At the functional level, CCA confirmed a global association between functional genes (KO) and the metabolome (Figure 12A). Spearman correlation analysis further delineated specific gene-metabolite regulatory axes (Figure 12B). Notably, the strongest positive correlation was observed between K20381 and desthiobiotin (*r* = 0.403, *P* < 0.001). Additionally, K02133 exhibited a significant positive correlation with gamma-glutamylvaline and a negative correlation with daidzein sulfate, while K25142 showed a specific positive association with L(-)-carnitine (Figure 12F).

Variable importance analysis identified Gene96 (K02027) as the most critical feature for classification (Figure 12C). The results of 10-fold cross-validation (Figure 12D) show that the fecal metabolomics model had the highest discriminatory ability (AUC = 0.704), followed by the metagenomic functional model (AUC = 0.609), while the combined metabolomics and functional gene model had an AUC of 0.631. Permutation testing of the combined nested model showed an empirical *P*-value of 0.0647 with a 95% CI of 0.491–0.744, which did not reach the conventional level of statistical significance, suggesting that this combined model did not demonstrate superior discriminatory ability compared with the single-omics models.

**Figure 13.**
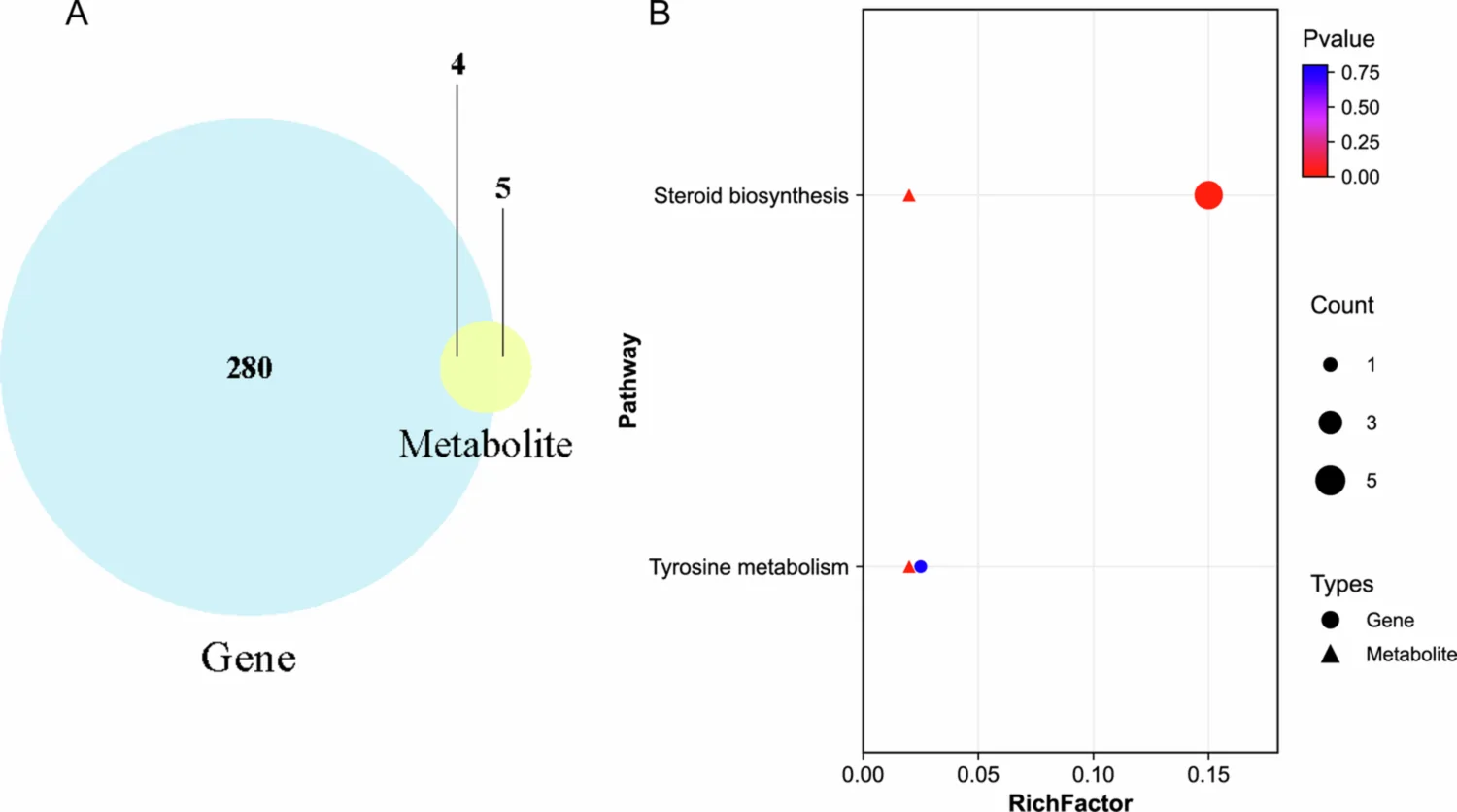
Combined biofunctional analysis of fecal metabolomics and metagenomics. (A) Venn diagram of pathways. This displays the overlap between differentially metagenomic genes and metabolites at the KEGG pathway level. (B) Bubble chart of pathway enrichment analysis. The x-axis represents the enrichment factor (RichFactor), where a higher value indicates a greater proportion of differentially annotated metabolites and genes assigned to that pathway. Circles denote **differential metagenomic genes**, while triangles represent metabolites. Shape size reflects the number of **differential metagenomic genes** assigned to the pathway, and color indicates pathway significance.

To further clarify the correspondence between metagenomic functional changes and metabolite alterations in biological pathways, this analysis combined the screening of differentially functioning genes (KOs) and differentially functioning metabolites using the KEGG database. The Venn diagram (Figure 13A) shows the intersection of the two omics at the pathway level. The metagenomic data involved 273 pathways, and the metabolomics data involved 10 pathways, with 11 shared pathways between the two. Bubble plot analysis (Figure 13B) further highlighted two key shared pathways: steroid biosynthesis and tyrosine metabolism. Interestingly, these pathways exhibited distinct enrichment patterns. Steroid biosynthesis was predominantly altered at the metagenomic level, as evidenced by larger, darker red circles, whereas metabolite enrichment was minimal. In contrast, tyrosine metabolism was significantly enriched at the metabolite level (red triangles), despite lower significance at the gene level (blue circles). The co-enrichment of these two pathways suggests that “Steroid biosynthesis” and “Tyrosine metabolism” may be key pathways linking gut microbiota dysfunction to changes in the host's metabolic phenotype.

#### 3.5.2. Systemic association between gut microbiota and serum metabolites

**Figure 14.**
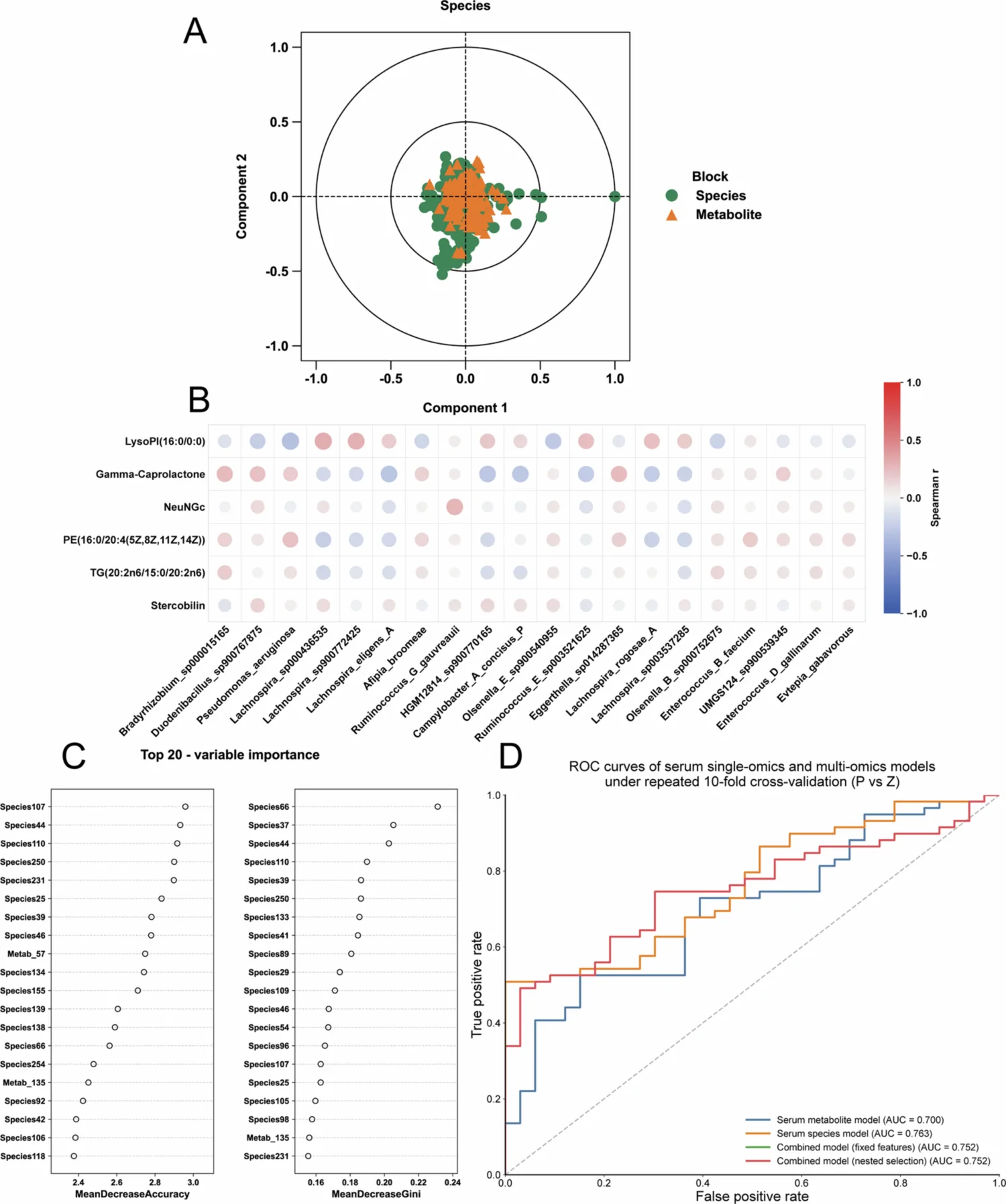
Multi-omics association analysis of gut microbiota and serum metabolites at the species level. (A) Canonical correlation analysis (CCA) plot. Green dots represent metagenomic species, and orange triangles represent metabolites. The correlation coefficient of the inner small circle is 0.5. The correlation coefficient of the outer large circle is 1. (B) Spearman correlation heatmap between differentially species (rows) and differentially metabolites (columns). The color from blue to red indicates that the correlation coefficient is from negative to positive. The significance level is marked as *P < 0.05 and **P < 0.01. (C) Variable importance analysis plot based on a random forest classifier. The left figure is the mean decrease accuracy, and the right figure is the mean decrease Gini index. The larger the value, the higher the importance of the variable in distinguishing PSD status. (D) The ROC curves based on the random forest model were internally validated using 10-fold cross-validation. The figure shows the discriminatory performance of the serum metabolite model, the serum species model, the fixed-feature combined model, and the nested-feature screening combined model, with AUC values of 0.700, 0.763, 0.752, and 0.752, respectively.

To elucidate the systemic interplay between gut microbiota and host metabolism, we performed an integrated analysis of metagenomic species and serum untargeted metabolomics data. Canonical correlation analysis (CCA) revealed significant covariation patterns in the first two canonical dimensions, confirming a robust global association between the two omics layers (Figure 14A). Spearman correlation analysis further delineated specific genus-metabolite interactions (Figure 14B). Notably, the genus *Lachnospira* exhibited the strongest metabolic associations. Several strains, including *L. eligens* A, *L. rogosae* A, and *L.* sp003463535, were positively correlated with LysoPI(16:0/0:0) (*r* > 0, *P* < 0.05) but negatively correlated with gamma-caprolactone and PE(16:0/20:4) (*P* < 0.05, blue cells). In stark contrast, *Pseudomonas aeruginosa* displayed an inverse pattern: a significant negative correlation with LysoPI(16:0/0:0) and a positive correlation with PE(16:0/20:4). However, after applying the Benjamini-Hochberg multiple correction to all correlation tests, none of the above associations reached statistical significance (FDR < 0.05) (Figure 14B, Supplementary Table 9).

Random forest analysis identified two core biomarkers based on MeanDecreaseAccuracy and MeanDecreaseGini: Species107 (*Enterococcus B faecium*) and Species66 (*HGM12814* sp900770165). These microbial features served as key discriminators between depression and non-depression groups (Figure 14C). Under 10-fold cross-validation (Figure 14D), the AUCs for the serum metabolite model and the serum species model were 0.700 and 0.763, respectively, while the AUC for the combined model was 0.752 under both the fixed-feature and nested-feature selection strategies. Further permutation tests showed that the combined nested model had an empirical *P*-value of 0.004975 with a 95% CI of 0.661–0.848, suggesting that its overall performance was significantly better than chance. However, the discriminatory ability of the combined model was still slightly lower than that of the serum species omics model, indicating that direct integration of serum metabolites and species-level data provides limited predictive gain for PSD classification.

**Figure 15.**
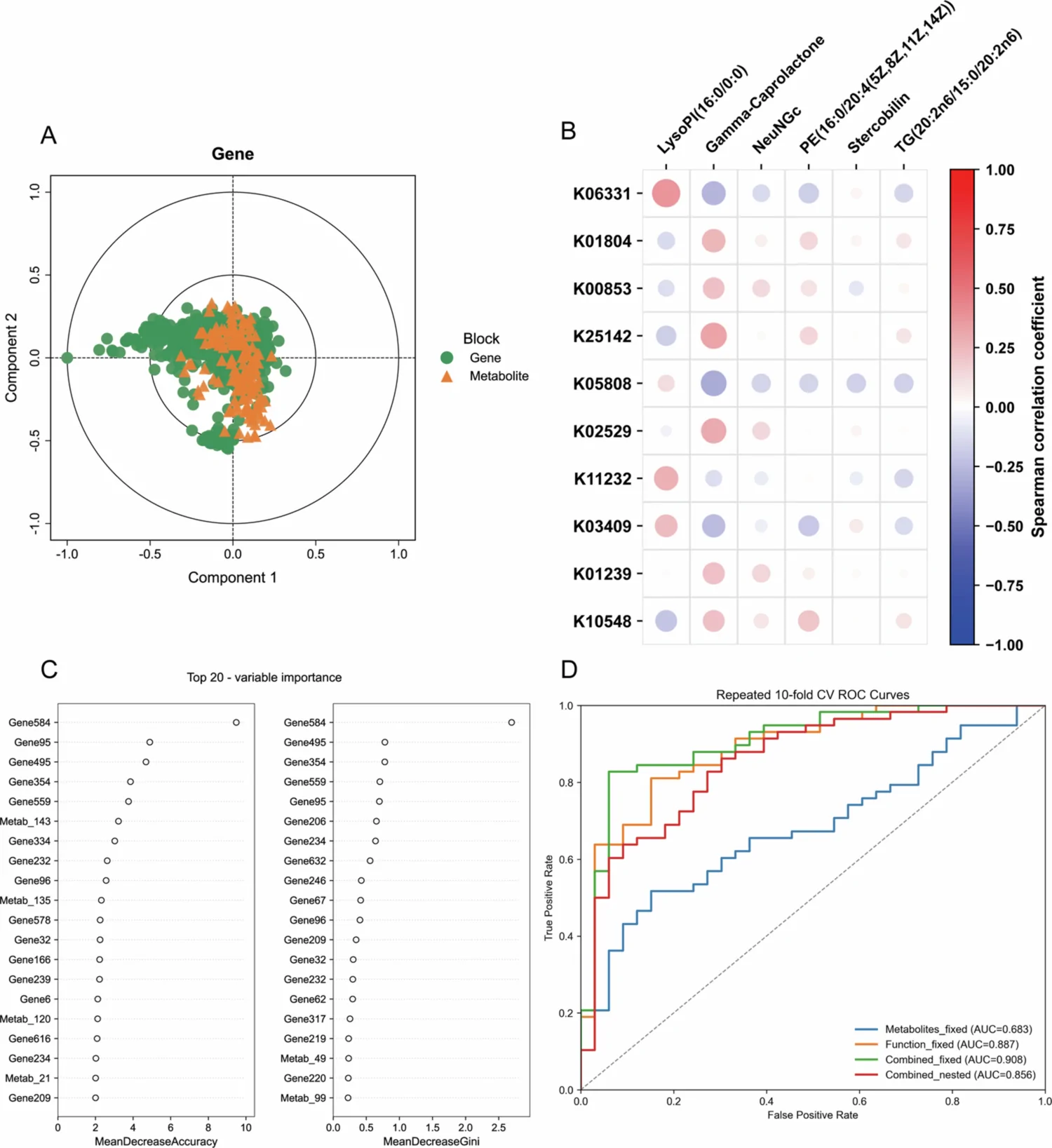
Multi-omics correlation analysis between gut microbiota and serum metabolites at the functional gene level. (A) Canonical correlation analysis (CCA) plot. Green dots represent metagenomic functional genes, while orange triangles denote metabolites. The inner circle with small dots indicates a correlation coefficient of 0.5. The outer circle with large dots indicates a correlation coefficient of 1. (B) Spearman correlation heatmap between differentially functional genes (rows) and metabolites (columns). Colors ranging from blue to red indicate correlation coefficients from negative to positive. Significance levels are marked as *P < 0.05 and **P < 0.01. (C) Variable importance analysis based on the random forest classifier. The left panel shows Mean Decrease in Accuracy (MDAcc), and the right panel shows Mean Decrease in Gini (MDGini). Higher values indicate greater importance of the variable in distinguishing PSD states. (D) ROC curves for the serum metabolite model, metagenomic functional model, and combined model under a 10-fold cross-validation framework. The blue line represents the serum metabolite model, the green line represents the metagenomic functional model, the orange line represents the combined model built using pre-selected candidate features, and the red line represents the results of a nested analysis in which feature selection and model training were performed within each training fold of the combined model. The aggregated AUCs for each model were 0.683, 0.887, 0.908, and 0.856, respectively.

At the functional gene (KO) level, the CCA results (Figure 15A) revealed distinct co-aggregation patterns. Differential KOs clustered predominantly along the negative axis of the first canonical variable, whereas differential metabolites were tightly grouped around the origin and positive axis. Further Spearman correlation analysis (Figure 15B; Supplementary Table S10) revealed several associations between differential KOs and serum metabolites. Among them, K06331 and K11232 generally exhibited similar metabolic association trends, both showing a positive correlation with the serum lipid metabolite LysoPI(16:0/0:0); K25142, K02529, and K01804 showed a positive correlation with gamma-caprolactone, whereas K06331, K05808, and K03409 exhibited a negative correlation with gamma-caprolactone. Overall, this suggests that different functional genes may correspond to metabolic changes in different directions. However, after Benjamini–Hochberg correction for multiple comparisons, none of the aforementioned functional gene–metabolite correlations reached statistical significance.

Random forest analysis identified Gene584 (K19545) as a potentially important feature for distinguishing between PSD and NPSD (Figure 15C). In a rigorous 10-fold cross-validation, the AUCs of the serum metabolite model, the metagenomic functional model, and the combined model were 0.683, 0.887, and 0.908, respectively; after applying nested feature selection within each fold in the combined model, the aggregated AUC was 0.856 (Figure 15D). This suggests that, in the current dataset, the integration of serum metabolites and metagenomic functional features under a fixed-feature strategy can improve discriminatory performance; however, this gain is somewhat diminished after more rigorous nested feature selection. The empirical *P*-value from the permutation test for the combined model was 0.004975, indicating that indicating that it performed significantly better than chance.

**Figure 16.**
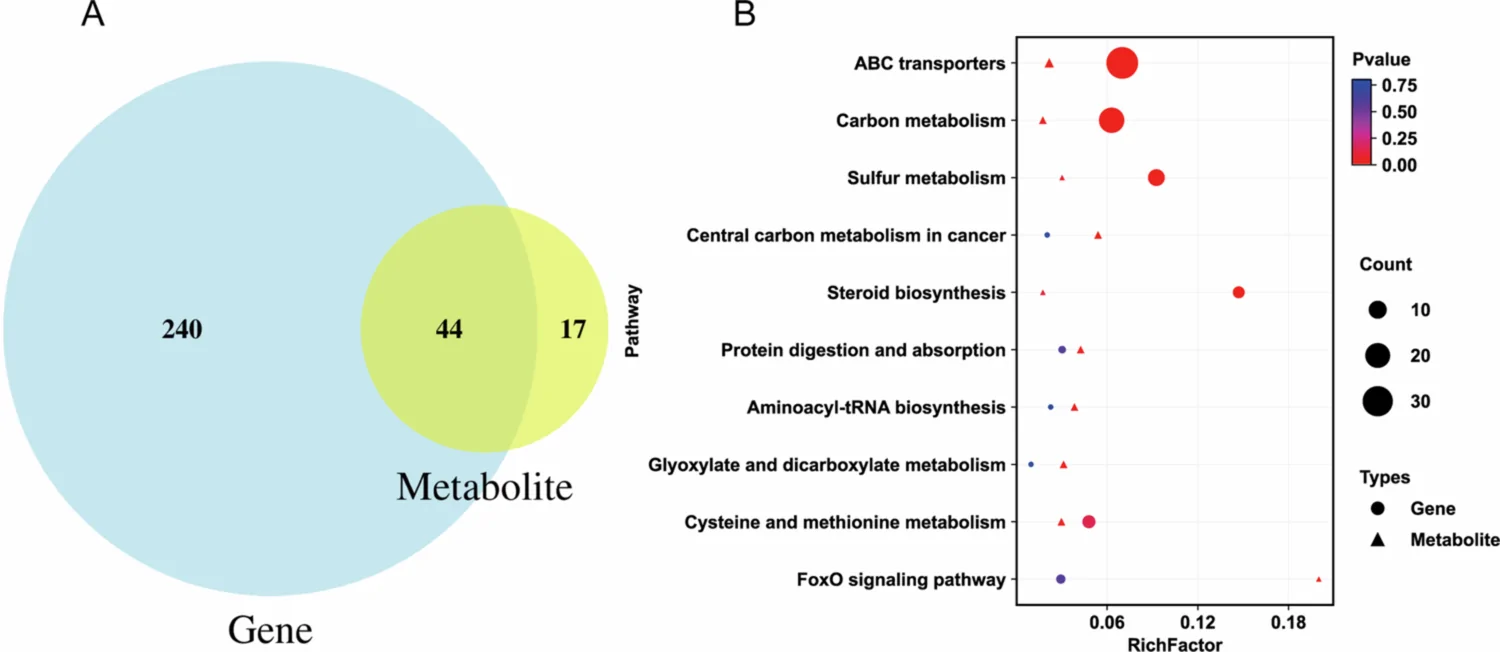
Integrated biofunctional analysis of serum metabolomics and metagenomics. (A) Venn diagram of pathways. This displays the overlap between differentially metagenomic genes and metabolites at the KEGG pathway level. (B) Bubble chart of pathway enrichment analysis. The x-axis represents the enrichment factor (RichFactor); a higher value indicates a greater proportion of differentially annotated metabolites and genes assigned to that pathway. Circles denote **metagenomic functional genes**, while triangles represent metabolites. Shape size reflects the number of differentially annotated indicators assigned to the pathway, and color indicates pathway significance.

Joint KEGG pathway analysis was performed to elucidate the biological mechanisms underlying these associations. The Venn diagram identified 44 metabolic pathways shared between serum metabolomics and metagenomics (Figure 16A). Bubble plots further illustrated the distinct distribution of these enriched pathways (Figure 16B). Differential functional genes in the metagenome were primarily enriched in “ABC transporters” and “Carbon metabolism”, with ABC transporters showing the highest gene count. In contrast, differential serum metabolites were predominantly enriched in the “FoxO signaling pathway” and neurotransmission-related pathways.

#### 3.5.3. Correlation analysis between significantly different bacteria and key metabolites

Based on the above results, we further identified six key metabolites (indolelactic acid, phenyllactic acid, LysoPI(16:0/0:0), Pivaloylcarnitine, gamma-glutamylvaline, and GABA) and five differentially abundant bacterial taxa (*Evtepia*, *Lachnospira*, *Blautia A*, *Alistipes*, and *Evtepia_gabavorous*) (including four genus-level taxa and one species-level taxon). Spearman's correlation analysis was performed, and FDR correction was applied using the Benjamini-Hochberg method. The results showed that, among the 30 microbiota-metabolite associations, 6 pairs remained statistically significant after FDR correction. Specifically, Evtepia_gabavorous was negatively correlated with phenyllactic acid (*r* = −0.365, q = 0.01128), Blautia A was negatively correlated with GABA (*r* = −0.341, q = 0.01307), Evtepia_gabavorous was negatively correlated with indolelactic acid (*r* = −0.332, q = 0.01307), Lachnospira was positively correlated with LysoPI(16:0/0:0) (*r* = 0.318, q = 0.01587), Evtepia was negatively correlated with phenyllactic acid (*r* = −0.303, q = 0.01821), and Lachnospira was positively correlated with gamma-glutamylvaline (*r* = 0.302, q = 0.01821) (Table 3).

**Table 3.** FDR-significant results from Spearman correlation analysis between key metabolites and differentially abundant taxa.

Metabolite	Taxon	N	Spearman	Raw P	FDR-adjusted q	Direction
Phenyllactic acid	Evtepia_gabavorous	91	−0.365	0.000376	0.011278	Negative
GABA	Blautia_A	91	−0.341	0.000953	0.013072	Negative
Indolelactic acid	Evtepia_gabavorous	91	−0.332	0.001307	0.013072	Negative
LysoPI(16:0/0:0)	Lachnospira	91	0.318	0.002116	0.015867	Positive
Phenyllactic acid	Evtepia	91	−0.303	0.003489	0.018209	Negative
Gamma-glutamylvaline	Lachnospira	91	0.302	0.003642	0.018209	Positive

Note: Spearman's rank correlation analysis was performed to assess the associations between 6 key metabolites and 5 differentially abundant taxa, yielding 30 pairwise comparisons in total. False discovery rate (FDR) correction was performed using the Benjamini–Hochberg method. Only associations that remained statistically significant after FDR correction are shown. *N*, sample size; Spearman, Spearman correlation coefficient; Raw *P*, unadjusted *P* value; FDR-adjusted q, q value after FDR correction; Direction, direction of association.

## 4. Discussion

Through a multi-omics integrated analysis, this study systematically characterized changes in the gut microbiota, fecal and serum metabolomics, and host inflammatory status in patients with post-stroke depression (PSD) of varying severity. Overall, the severity of PSD was associated with coordinated changes in gut microbiota composition, host metabolic remodeling, and inflammation-related features.

### 4.1. Alterations in gut microbiota structure and clinical characteristics

Most studies report reduced gut microbiota diversity in depressed patients. However, this study observed significantly higher Shannon and Simpson indices in patients with post-depressive disorder (PSD), especially those with moderate depression, compared to the non-depressed group. This increased diversity does not necessarily indicate a healthy ecosystem; it may suggest an overgrowth of opportunistic pathogens or non-dominant bacteria, disrupting the original gut balance. This phenomenon has also been reported in recent PSD studies, suggesting that the relationship between gut microbiota diversity and disease severity may be more complex.^8^

Through metagenomic analysis, this study revealed a significant gradient correlation between the degree of gut microbiota dysbiosis and the severity of depressive symptoms in PSD patients. Regarding microbial composition, we observed an increased abundance of the phylum *Firmicutes* and a decreased abundance of Bacteroidota; Firmicutes/Bacteroidota (F/B) ratio has frequently been associated with metabolic abnormalities in previous studies. A study by Parker et al. reported that *Alistipes* may influence tryptophan metabolism through indole metabolite production, potentially affecting intestinal barrier integrity and neuroinflammatory processes.^9^ In the present study, the abundance of *Alistipes* in this study was positively correlated with key inflammatory markers such as IL-6, IL-1β, and TNF-*α*, suggesting that it may be involved in the PSD-associated inflammatory phenotype; however, these correlations were not significant after multiple adjustments and are better suited as hypothesis-generating clues. Whether *Alistipes* plays a causal role in PSD remains unclear. Meanwhile, *Bacteroides* is a Gram-negative genus and a potential source of lipopolysaccharide (LPS), which is associated with host immune activation. Recent literature has also suggested that microbiota-associated molecular patterns may amplify inflammatory responses through host innate immune pathways (cGAS-STING), providing mechanistic context for the “gut microbiota–immune activation–neuroinflammation” pathway.^10^ Impaired intestinal barrier function following stroke may facilitate LPS translocation into the bloodstream, which may contribute to neuroinflammation.^5^ Together with the enrichment of LPS-related functional modules observed in this study, these findings suggest that a complex interplay may exist between gut microbiota dysbiosis and host inflammatory signaling in PSD. Furthermore, *Blautia*, a beneficial bacterium, produces short-chain fatty acids (SCFAs) that exert anti-inflammatory effects and help maintain the intestinal barrier^11^; potentially reflecting a compensatory host response.

In this study, no broadly consistent correlation patterns were observed between differential microbial communities and key metabolites; instead, relatively distinct, specific, and selective associations between microbial communities and specific metabolites were observed. First, the negative correlation between *Blautia A* and GABA remained significant even after incorporating multiple representative metabolites and applying unified FDR correction, suggesting that it may be a relatively robust microbial signal among metabolic abnormalities associated with PSD-related neuroactivity. In contrast, the negative correlation between Evtepia and its species-level member Evtepia gabavorous and GABA did not remain significant after FDR correction, indicating that the current data do not provide sufficient support for considering them key microbial contributors to GABA-related alterations. Previous studies, including more recent reports, have suggested that GABA does not readily cross the blood–brain barrier (BBB) to enter the brain; therefore, GABA levels in peripheral blood often may not directly reflect the neurotransmitter state within the central nervous system.^12^ Furthermore, Chen et al. (2021) also emphasized that for neuroactive substances unable to freely cross the BBB, the vagus nerve may represent an important pathway through which such substances exert central regulatory effects.^13^ Existing research suggests that peripheral inflammatory factors may exert indirect regulatory effects on distal tissues through complex immune-metabolic networks. A review by Wang et al. on IL-6 also suggests that the biological effects of peripheral inflammatory signals are not limited to local tissues but may influence systemic functions through various indirect pathways.^14^ In summary, given the limited ability of GABA to cross the blood–brain barrier, the results of this study suggest that *Blautia A*-related changes in GABA metabolism may influence gut-brain communication through indirect pathways—such as the vagus nerve, immune regulation, and alterations in intestinal barrier/blood-brain barrier function—and may therefore be associated with depressive symptoms.

It is worth noting that *Evtepia_gabavorous* showed negative correlations with both phenyllactic acid and indolelactic acid, while the genus *Evtepia* also exhibits a significant negative correlation with phenyllactic acid. This consistency at the genus-species level suggests that the metabolic associations of *Evtepia*-related microbiota in PSD may be more closely linked to aromatic amino acid- and indole-related metabolic pathways than to a single GABA-related pathway. Previous studies have shown that microbiota-derived aromatic amino acid metabolites can participate in gut–brain axis communication by regulating immune and inflammatory responses, maintaining intestinal barrier homeostasis, and influencing neural signal transduction.^15^ On the other hand, the positive correlation between *Lachnospira* and LysoPI(16:0/0:0) and gamma-glutamylvaline suggests that it may be more closely associated with lipid metabolism and alterations in the amino acid-related metabolic environment. Phosphatidylinositol-related lipid metabolism is linked to membrane signaling, inflammatory responses, and neuronal homeostasis, while gamma-glutamylvaline is generally considered to be associated with metabolic networks related to amino acid turnover and oxidative stress. Therefore, these findings suggest that gut microbiota dysbiosis associated with PSD may not be driven by a single metabolic axis, but may instead involve multiple relatively independent yet interconnected microbiota–metabolite modules.

### 4.2. Differences in metabolic characteristics across different severity levels of depression

The mild and moderate PSD subgroups showed distinct enrichment patterns in fecal and serum metabolic profiles, suggesting that different severity levels of depression may correspond to different systemic metabolic states.

In the Mild-PSD group, there was an higher fecal enrichment of acetylcholine was observed in feces and related pathways, such as cholinergic synapses and synaptic vesicle cycle, which may reflect compensatory activation of the vagus nerve-gut-brain axis. As a core molecule in the cholinergic anti-inflammatory pathway, the elevation of acetylcholine may be an attempt by the body to inhibit the release of pro-inflammatory factors through activation of α7nAChR (α7 nicotinic acetylcholine receptors).^16^ This upregulation may be a self-protective mechanism of the body in the face of early inflammatory stress, possibly accompanied by inhibition of energy metabolism. This compensatory mechanism may inhibit core energy pathways such as the tricarboxylic acid (TCA) cycle in serum, which could be relevant to symptoms such as fatigue and anhedonia.^17^ Furthermore, elevated levels of lysophospholipids such as LysoPI suggest alterations in cell membrane stability, indicating a potential association with lipid metabolism disorders and changes in membrane homeostasis, and may provide a metabolic backdrop for damage related to lipid peroxidation.

In the Mod-PSD group, a dual enrichment of tryptophan metabolism and ABC transporters was observed in both fecal and serum samples. The significant accumulation of ceramide and sphingolipids in serum suggests that moderate PSD may be associated with lipid metabolism disorders and changes related to oxidative stress. Other studies have reported that ceramides may cross the blood-brain barrier and have been associated with neuronal apoptosis and reduced brain-derived neurotrophic factor (BDNF) expression, potentially contributing to depressive symptoms.^18^

From a mechanistic perspective, lipid peroxidation may be a key underlying factor in oxidative damage in the moderate PSD group. Neuronal cell membranes are rich in polyunsaturated fatty acids, making them susceptible to reactive oxygen species-mediated attack and chain-propagating lipid peroxidation, which in turn disrupt membrane structural integrity and exacerbate neuronal damage; iron dyshomeostasis may play a key amplifying role in this process. In recent years, a growing body of research has demonstrated that this iron-dependent lipid peroxidation damage may have important pathological relevance in neurological diseases and ischemia-reperfusion injury. Ferroptosis can be understood as a form of cellular damage characterized by the accumulation of iron-dependent lipid peroxides. Relevant studies suggest that GPX4 is one of the key antioxidant enzymes that inhibits this process by maintaining lipid redox homeostasis through the scavenging of membrane phospholipid hydroperoxides.^19^ When considered together with the antioxidant-related metabolic abnormalities, lipid metabolism disorders, and enrichment of ferroptosis-related pathways observed in this study, these findings suggest that the moderate group may have a metabolic background conducive to membrane lipid peroxidation and redox imbalance, potentially accompanied by the involvement of iron-related damage pathways. Furthermore, previous studies have suggested the existence of a potentially self-reinforcing pathological loop between neuroinflammation and lipid peroxidation/ferroptosis: inflammatory factors and oxidative stress may further exacerbate iron metabolism disorders and lipid peroxidation, while the resulting cellular damage may in turn promote local inflammatory responses.^20^ However, since this study did not directly measure GPX4, ACSL4, SLC7A11, iron ion load, or lipid peroxidation end products, the above explanation remains a potential mechanistic framework proposed based on multi-omics results and requires further experimental validation.

Imbalances in tryptophan metabolism, particularly the depletion of serotonin (5-HT), may be related to the pathophysiology of PSD.^21^ Since systemic tryptophan availability influences central 5-HT synthesis,^22^ its deficiency may affect the regulation of mood, sleep, and pain processing.^23^ In addition, the post-stroke inflammatory cascade may also reduce the bioavailability of 5-HT by enhancing the metabolic shunting of the kynurenine pathway.^24^ Multiple studies have reported that inflammatory responses or gut microbiota dysbiosis may induce upregulation of indoleamine 2,3-dioxygenase (IDO), thereby shifting tryptophan metabolism toward the kynurenine pathway and increasing the production of cytotoxic quinolinic acid, which has been associated with depression.^25^ However, after systematic re-evaluation in the present study, the currently observed tryptophan-related abnormalities were mainly concentrated in the indole-related branch, whereas evidence for alterations in the kynurenine and 5-HT branches remained limited (metabolite-level re-screening results are provided in Supplementary Tables S4 and S5, and the branch-level summary is provided in Supplementary Table S6). Therefore, these findings are better regarded as preliminary clues indicating that tryptophan metabolism may be disturbed in PSD, and further validation by targeted metabolomics is still needed. In addition, fecal 3-indoxyl sulfate is an indole-derived metabolite produced from tryptophan by gut microbiota. Previous studies in patients with chronic kidney disease have shown that this metabolite may induce oxidative stress, endothelial dysfunction, and neurotoxicity,^26^ further supporting a possible link between gut microbial alterations and host metabolic abnormalities.

Furthermore, the dual enrichment of ABC transporter pathways may reflect altered transport-related functions of gut microbiota-derived substances in PSD, potentially affecting the systemic availability of microbial neurotrophic precursor molecules (e.g., B vitamins, carnitine) into the circulation. This finding may reflect a potential link between functional changes in the gut microbiota and alterations in host metabolic status.^27^ ^,^ ^28^

Based on the observed metabolic dysregulation in PSD, this study further screened specific biomarkers for disease stratification, providing preliminary clues for severity classification. For example, PI(18:2/0:0) showed potential discriminatory value in distinguishing Mild-PSD from non-depressed controls (NPSD) (AUC = 0.732). As a representative phosphatidylinositol molecule, its altered level may be related to lipid metabolic disturbances and changes in membrane fluidity. In differentiating Mild-PSD from Mod-PSD, 2-Aminomuconic acid semialdehyde—an intermediate of the tryptophan-kynurenine pathway—exhibited robust discriminatory power between Mild-PSD and Mod-PSD (AUC = 0.795). Recent evidence underscores that abnormal activation of the kynurenine pathway not only involved in depression pathology but may also be closely associated with antidepressant treatment response.

In distinguishing Mod-PSD from NPSD, 5alpha-androstan-3alpha, 17alpha-diol monosulfate demonstrated relatively good discriminatory efficacy (AUC = 0.796). Previous studies have suggested that this compound may represent a sulfated metabolite of endogenous neurosteroids and may influence neuronal excitability through bidirectional allosteric modulation of GABA-A receptor function; its positive modulatory effects may be associated with anxiolytic and antidepressant actions.^29^ ^,^ ^30^ Our findings are generally consistent with previous studies on PSD pathogenesis. Several studies have suggested that ischemic stroke may disrupt the brain's glutamate/GABA (excitation/inhibition) balance, and impaired GABAergic transmission may be involved in the pathophysiology of PSD.^31^ In addition, previous studies have indicated that depression is associated with alterations in mitochondrial function and energy metabolism, and that neurosteroids may alleviate depressive states by promoting cellular energy production through glycolysis and respiratory-mediated energy metabolism.^32^ ^,^ ^33^ From a broader metabolic regulatory perspective, the downregulation of carbon metabolism-related pathways, energy metabolic remodeling, and stage-specific lipid metabolic abnormalities observed in this study may be related to dysregulation of cellular energy-sensing systems and mitochondrial homeostasis. Previous studies have shown that the AMPK/SIRT1/PGC-1α signaling axis may play an important role in coordinating mitochondrial biogenesis, oxidative stress defense, and inflammatory homeostasis.^34^ ^,^ ^35^ In this context, the metabolic differences observed between the Mild-PSD and Mod-PSD groups may reflect differences in adaptive metabolic regulation and redox buffering capacity across severity levels. However, because this study did not directly assess AMPK, SIRT1, PGC-1α, or mitochondrial function-related indicators, these interpretations should be viewed as indirect theoretical explanations for the observed metabolic features rather than direct evidence. In addition, the post-stroke peripheral inflammatory milieu may indirectly contribute to central excitation/inhibition imbalance by altering blood-brain barrier homeostasis, activating microglia, or affecting astrocyte-mediated local GABA metabolism. These findings suggest that this metabolite may be associated with PSD severity and may represent a candidate indicator of post-stroke neuroactive metabolic alterations.

The above analysis suggests that serum metabolites exhibit higher sensitivity and specificity in reflecting the progression of PSD. This may be because the serum metabolome more directly reflects the body's systemic homeostasis and the overall function of the brain-body metabolic axis, compared to factors influenced by the gut environment.^36^ While fecal biomarkers possess some discriminative power, their overall diagnostic efficacy (AUC < 0.78) is slightly lower than that of serum biomarkers. These results suggest that serum metabolic biomarkers, in combination with fecal metabolic features, may be worth further exploration in future PSD stratification models.

### 4.3. Cross-omics correlation analysis of gut microbiota and host metabolism

The association analysis between the microbiome and metabolome provides a global perspective for understanding the pathological mechanisms of PSD. This study, through the association analysis of metagenomics and metabolomics, revealed a complex “host-microbe” metabolic interaction network, suggesting that gut microbiota alterations may be linked to changes in host physiological status through associations with specific metabolites.

#### 4.3.1. Impaired gut barrier function and systemic inflammatory response

Association analysis of serum metabolites and gut microbiota suggested that, compared with species-level abundance, metagenomic functional features might provide additional PSD-related discriminatory information in serum-based joint modeling. After repeated 10-fold cross-validation with nested feature selection within each fold, the aggregated AUC of the joint model decreased to 0.856, indicating that the integration of serum metabolites and metagenomic functional features may have exploratory auxiliary discriminatory value in the current cohort, but at this stage it may be more appropriately regarded as providing supportive clues for identification rather than as a clinically generalizable diagnostic model.

At the species level, *Lachnospira* -related taxa showed a positive correlation trend with the serum lipid metabolite LysoPI(16:0/0:0). As a product of phospholipase A2-mediated phospholipid hydrolysis, *has been associated with inflammatory status.* ^37^ Although *Lachnospira* is a known short-chain fatty acid (SCFA) producer whose depletion is typically linked to lipid dysregulation, its association with inflammation here may reflect compromised intestinal barrier function rather than intrinsic pathogenicity.^38^ Previous studies have suggested that intestinal barrier integrity plays an important role in preventing gut pathogens and toxins from entering the systemic circulation.^39^ Impairment of the intestinal barrier has also been associated with changes in gut microbial composition and metabolite profiles. In this context, systemic inflammatory responses after stroke may be related to impaired intestinal barrier function, reduced SCFA levels, bacterial translocation, and the entry of LPS into the bloodstream. Under conditions of barrier dysfunction, metabolites derived from the luminal microbiota may be more likely to enter the circulation more readily together with endotoxins, thereby showing synchronous increases with systemic inflammatory markers. Relevant reviews have suggested that gut barrier dysfunction and bacterial translocation are important mechanisms linking environmental factors, metabolic stress, and systemic inflammation, and that SCFAs may alleviate PSD-related neuroinflammation by maintaining blood-brain barrier (BBB) integrity and reducing the entry of inflammatory factors.^40^ ^,^ ^41^ Taken together with our findings, these observations suggest that post-stroke alterations in intestinal barrier function, reduced SCFA levels, bacterial translocation, and LPS influx into the circulation may jointly contribute to the formation of a systemic inflammatory milieu.

#### 4.3.2. Microbial nutritional competition and antioxidant metabolic imbalance

In the local gut microenvironment, Desthiobiotin was identified as the most highly connected node in the network, showing significant associations with 18 microbial species. As a key precursor in biotin (vitamin B7) biosynthesis, it showed significant positive correlations with *Bradyrhizobium*, *Pseudomonas*, and *Enterococcus faecium*, while being negatively correlated with *Lachnospira*. Biotin is an essential cofactor for neurotransmitter synthesis and energy metabolism, and the human gut microbiota is also an important source of biotin for the host. Our findings suggest that the expansion of *Enterococcus* may be associated with abnormal accumulation of desthiobiotin in the gut, potentially through competition for biosynthetic substrates or interference with precursor conversion, thereby potentially contributing to a relative insufficiency of active biotin. Such an imbalance between precursor and end-product metabolites may affect mitochondrial energy metabolism and carboxylation reactions required for nervous system function, and may therefore affect processes involved in maintaining neuronal function.

Furthermore, fecal *Enterococcus type B* showed a significant negative correlation with *γ*-glutamylvaline. This negative association further suggests that the expansion of this bacterium may be accompanied by reduced local intestinal antioxidant capacity, which is consistent with studies reporting dysregulation of glutathione metabolism in patients with major depressive disorder,^42^ and glutathione has been proposed as a potential biomarker for early depression.^43^ Taken together, these observations suggest that expansion of this taxon may be associated with reduced host antioxidant capacity.

KEGG-based integrative pathway analysis further revealed potential gut-brain axis-related signaling features. Metagenomic functions were mainly enriched in the ABC transporter pathway, whereas serum metabolites were enriched in the FoxO signaling pathway. This pattern suggests that gut microbiota may influence the systemic distribution of bioactive molecules through transport-related functions and may potentially be associated with FoxO pathway-related signaling in distal tissues, including the nervous system. Previous studies have shown that the FoxO pathway may play an important role in regulating neuronal oxidative stress, autophagy, and apoptosis.^44^ ^,^ ^45^ For example, activation of the *p*-Foxo3a/CREB signaling pathway has been reported to be involved in the neuroprotective effect of Swell1 and to alleviate neurological dysfunction caused by ischemic stroke. However, as this study did not directly assess FoxO signaling activity or its downstream molecules, its specific mechanistic role in PSD remains unclear.

**Figure 17.**
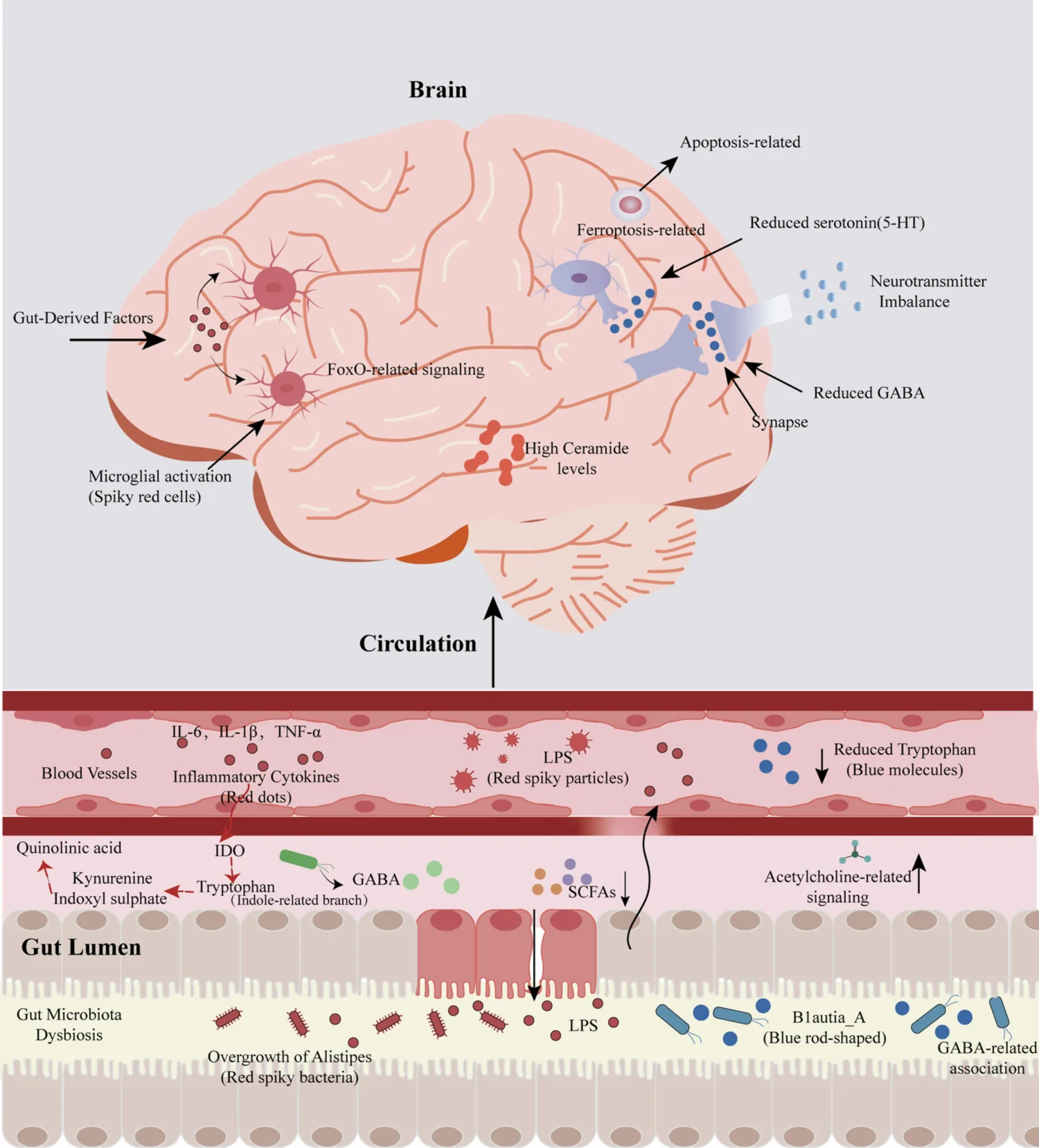
A hypothetical model of gut microbiota-related metabolic abnormalities and host neuroinflammatory changes in post-stroke depression (PSD). This figure was constructed based on the multi-omics association results from this study, combined with previous literature, to summarize the potential “gut microbiota dysbiosis–metabolic remodeling–systemic inflammation–brain functional changes” framework in PSD. The diagram is divided into three levels: the intestinal lumen, the circulation, and the brain. At the intestinal lumen level, PSD patients may exhibit a microbiota–metabolic remodeling characterized by Alistipes enrichment, Blautia_A and GABA-related changes, reduced short-chain fatty acids, tryptophan-related metabolic abnormalities, and changes in cholinergic signaling; specifically, the mild stage of PSD may be more characterized by cholinergic anti-inflammatory compensation, whereas the moderate stage may be more marked by lipid metabolism disorders, elevated ceramides, and ferroptosis-related changes. These peripheral changes may be accompanied by impaired intestinal barrier function and the entry of LPS and pro-inflammatory cytokines into the bloodstream, and may further be linked to intracerebral changes such as microglial activation, FoxO-related signaling alterations, neurotransmitter homeostasis imbalance, and apoptosis/ferroptosis. The arrows in the figure indicate possible association pathways rather than causal relationships directly confirmed by this study; some of these links (dashed arrows) are based solely on indirect evidence or inferences from the literature and require further validation through longitudinal studies and mechanistic experiments. Note: The associations between the pathways shown in the figure are hypothetical frameworks and do not represent direct causal relationships. As this study employed a cross-sectional design, the chronological sequence of the aforementioned changes cannot be determined.

These observations suggest that gut microbiota dysbiosis in PSD, such as the enrichment of *Alistipes* and *Bacteroides*, may be associated with intestinal barrier disruption, systemic inflammation, and metabolic disturbances, including shifts in tryptophan metabolism and lipid peroxidation, thereby potentially contributing to processes related to central nervous system injury (Figure 17). Notably, the multi-omics features were not fully consistent across different depression severity strata. Compared with the acetylcholine-related metabolic features observed in the Mild-PSD group, the Mod-PSD group showed more evident ceramide accumulation, lipid metabolic dysregulation, oxidative stress-related alterations, and enrichment of ferroptosis-related pathways. These findings suggest that different PSD severity levels may correspond to distinct metabolic and functional features; however, given the cross-sectional design of this study, such differences should not be interpreted as a continuous temporal progression. Overall, the integrated multi-omics results are consistent with the hypothesis that PSD-related gut microbial alterations, host metabolic abnormalities, and inflammatory features may be interconnected. These findings provide clues for understanding the systemic pathological features of PSD and identifying potential stratification biomarkers, but further longitudinal and mechanistic studies are still needed.

## 5. Conclusion

Based on the integrative analysis of metagenomics, fecal metabolomics, and serum metabolomics, this study characterized the “microbiota-metabolism-inflammation” features of patients with post-stroke depression across different severity levels. Unlike previous studies that were largely limited to descriptive analyzes at a single time point or within a single omics layer, this study not only observed marked gut microbial dysbiosis in PSD, but also further suggested that such dysbiosis may be accompanied by functional remodeling across disease severity. In the mild stage, the metabolic pattern was characterized by enrichment of cholinergic-related anti-inflammatory pathways, whereas the moderate stage showed more prominent features of lipid peroxidation, ferroptosis-related metabolic alterations, and tryptophan metabolism dysregulation. These cross-sectional findings suggest the possibility of a shift from compensatory activation to functional imbalance. Overall, this study helps address the current lack of severity-stratified comparisons in PSD and provides support for the hypothesis that PSD pathology may not follow a single linear process, but instead involves a non-linear pathological process that may be shaped by multi-omics interactions.^46^

In the discrimination model analysis, the performance of different multi-omics combinations showed heterogeneous performance. Some integrated models, particularly the combinations of fecal species with fecal metabolites and serum metabolites with metagenomic functional features, showed better or potentially showed relatively better discriminatory performance than single-omics models. However, this gain was not consistently observed across all omics combinations, and after more stringent nested feature selection within the training folds, the performance of some integrated models was attenuated. These findings are generally consistent with recent trends in multi-omics integration research and provide supportive evidence that multi-omics integration may improve discriminatory performance, although its practical value and underlying mechanisms still require further validation in independent external cohorts. Previous studies have suggested that integrating gut microbiome, metabolome, and host genome data may help reflect the multi-layered pathological features of PSD and provide partial clues for identifying biomarker combinations with higher specificity and sensitivity.^43^ Accordingly, our results further support the use of metabolic phenotypes as a plausible intermediate layer linking microecological alterations and neuropsychiatric symptoms, and may help promote a shift in PSD management from symptomatic treatment toward mechanism-informed precision intervention.

Despite the novel multi-omics insights provided by this study, several limitations should be acknowledged. First, as a cross-sectional study, we did not perform fecal microbiota transplantation, targeted strain intervention, metabolite supplementation or blockade experiments, or causal inference analyzes. Therefore, the temporal order and causal relationships among gut microbiota alterations, metabolic abnormalities, inflammatory status, and PSD severity could not be determined, and the findings should be regarded as exploratory and hypothesis-generating. Second, although we observed GABA-related, tryptophan-related, oxidative stress-related, and FoxO pathway-related features, we did not directly measure key mechanistic molecules such as kynurenine, quinolinic acid, 5-HT, GPX4, lipid peroxidation end products, or iron metabolism-related markers. Thus, mechanistic interpretations remain limited. In particular, the evidence regarding 5-HT was derived mainly from pathway annotation and enrichment analysis rather than direct targeted quantification. Re-screening of tryptophan-related abnormalities also suggested that is more consistent with indole-related disturbances, whereas support for the kynurenine and serotonin-related branches remains relatively limited. Third, after more stringent internal validation, the performance of the integrated machine learning models declined compared with that of the fixed-feature models. Given the relatively limited sample size and high feature dimensionality, the original performance estimates may have been subject to some optimism bias. Accordingly, the modeling results should also be considered exploratory and still require validation in larger samples and independent external cohorts. Finally, although stroke duration, lesion distribution, and some medication use were included and compared at baseline, and sensitivity analyzes were conducted for PPI and antidepressant use, dietary structure and nutrient intake were not systematically recorded. Residual confounding by diet therefore cannot be excluded. In addition, some results changed after excluding users of specific medications, suggesting that drug exposure may still affect the stability of certain findings. Overall, future studies should incorporate larger samples, multicenter designs, and longitudinal follow-up, with stricter control of potential confounders and independent validation. Causal inference approaches, such as Mendelian randomization, may also help evaluate the potential causal relationship between gut microbiota and PSD using larger-scale genetic data.

In summary, this study provides integrated multi-omics clues regarding severity-associated gut-brain axis alterations in PSD and proposes several candidate biomarkers of potential clinical relevance. These findings provide additional clues for understanding PSD pathology and may inform future diagnostic and therapeutic research. Future research may further explore targeted nutritional interventions (e.g., specific probiotics, prebiotics, or metabolic precursors) and pharmacological therapies (e.g., ferroptosis and acid sphingomyelinase inhibitors) to evaluate their potential relevance to PSD. Such studies may help advance evidence-based precision strategies for PSD prevention and treatment.

## Supplementary Material

Supplemental_Material__1_.docx

Supplemental_Material.docx

## Data Availability

The fecal and serum untargeted metabolomics datasets generated in this study have been deposited in Zenodo under embargo and are available via the following DOI(s): https://doi.org/10.5281/zenodo.19394192(fecal); https://doi.org/10.5281/zenodo.19388744(serum). The metagenomic raw sequence data generated in this study have been deposited in the Genome Sequence Archive (GSA) at the National Genomics Data Center, China National Center for Bioinformation/Beijing Institute of Genomics, Chinese Academy of Sciences, under accession number CRA040936 that are publicly accessible at https://ngdc.cncb.ac.cn/gsa The files will be made publicly accessible upon publication of this article.
