# Supplementary material for: Integrated metagenomic and metabolomic analysis identifies severity-specific inflammatory and metabolic signatures in post-stroke depression
Source: Gut Microbes. 2026 Sep 14;18(1):2726620. doi: 10.1080/19490976.2026.2726620 (PMC13577261; doi:10.1080/19490976.2026.2726620)
Supplement: Supplementary file 1 [file KGMI_A_2726620_SM1057.docx]

**Supplementary Material**

**Supplementary Table**

**Supplementary** **Table S1**. Sensitivity analyses after excluding PPI users and antidepressant users

| Variable | Full sample | Excluding PPI users | Excluding antidepressant users |
| --- | --- | --- | --- |
| TNF-α | NS | NS | Significant(P = 0.031) |
| IL-6 | NS | NS | Significant(P = 0.023) |
| IL-10 | NS | Significant(P = 0.028) | NS |
| N6,N6,N6-Trimethyl-L-lysine | Significant(P = 0.015) | Significant(P = 0.011) | NS |
| Indolelactic acid | Significant(P = 0.002) | NS | Significant(P = 0.028) |
| Phenyllactic acid | Significant(P < 0.001) | NS | Significant(P = 0.047) |
| LysoPI(16:0/0:0) | Significant(P = 0.006) | NS | NS |
| Pivaloylcarnitine | NS | Significant(P = 0.013) | NS |
| γ-Glutamylvaline | Significant(P = 0.026) | NS | NS |
| Evtepia | Significant(P < 0.001) | NS | Significant(P = 0.015) |
| Lachnospira | Significant(P < 0.001) | NS | Significant(P = 0.028) |
| Blautia_A | Significant(P = 0.003) | NS | NS |
| Alistipes | Significant(P = 0.022) | NS | NS |
| Roseburia | Significant(P = 0.022) | NS | NS |
| K02529 | Significant | NS | NS |
| K21471 | Significant | NS | NS |
| K02026 | Significant | Significant(P = 0.029) | Significant(P = 0.039) |
| K02025 | Significant | Significant(P = 0.027) | Significant(P = 0.047) |
| K05808 | Significant | Significant(P = 0.019) | Significant(P = 0.007) |
| K06871 | Significant | Significant(P = 0.045) | Significant(P = 0.006) |
| K10119 | Significant | Significant(P = 0.018) | NS |
| K10118 | Significant | Significant(P = 0.018) | NS |

**Note**：Abbreviations: PPI, proton pump inhibitor; NS, not significant.

Sensitivity analyses were conducted after excluding PPI users and antidepressant users, respectively. Only variables with statistical significance in at least one analysis are shown. Reduced sample size in the restricted analyses may have limited statistical power.

**Supplementary Table S2A**. Kruskal–Wallis test results for species-level alpha-diversity indices among the Mod-PSD, Mild-PSD, and NPSD groups

| Index | Kruskal–Wallis H | Kruskal–Wallis P |
| --- | --- | --- |
| Chao1 | 1.969 | 0.374 |
| Simpson | 7.057 | 0.029 |
| Shannon | 8.001 | 0.018 |

**Note**: Overall differences in species-level alpha-diversity indices were assessed using the Kruskal–Wallis rank-sum test.

**Supplementary Table S2B.** Pairwise Wilcoxon rank-sum test results with Benjamini–Hochberg correction for species-level alpha-diversity indices among the Mod-PSD, Mild-PSD, and NPSD groups.

| Index | Pairwise comparison | U statistic | Raw P | BH-adjusted P | Significant |
| --- | --- | --- | --- | --- | --- |
| Chao1 | Mod-PSD vs Mild-PSD | 500.0 | 0.2067 | 0.3742 | No |
| Chao1 | Mod-PSD vs NPSD | 523.5 | 0.2495 | 0.3742 | No |
| Chao1 | Mild-PSD vs NPSD | 492.5 | 0.8037 | 0.8037 | No |
| Simpson | Mod-PSD vs Mild-PSD | 565.0 | 0.0229 | **0.0343** | Yes |
| Simpson | Mod-PSD vs NPSD | 604.0 | 0.0189 | **0.0343** | Yes |
| Simpson | Mild-PSD vs NPSD | 530.0 | 0.8089 | 0.8089 | No |
| Shannon | Mod-PSD vs Mild-PSD | 585.0 | 0.0097 | **0.0283** | Yes |
| Shannon | Mod-PSD vs NPSD | 604.0 | 0.0189 | **0.0283** | Yes |
| Shannon | Mild-PSD vs NPSD | 503.0 | 0.9144 | 0.9144 | No |

**Note**: Pairwise comparisons were performed using the Wilcoxon rank-sum test. P-values were adjusted for multiple testing using the Benjamini–Hochberg method. Significant adjusted p-values are shown in bold.

**Supplementary Table S3A**. Spearman correlations between differential bacterial taxa and inflammatory markers in the study population.

| **Bacterial taxon** | **Inflammatory marker** | **N** | **Spearman r** | **Raw P** | **FDR-adjusted P** |
| --- | --- | --- | --- | --- | --- |
| Faecalibacterium | IL-10 | 91 | -0.307 | 0.0031 | 0.2141 |
| Alistipes | IL-1β | 91 | 0.268 | 0.0101 | 0.34 |
| Enterococcus_faecalis | IL-10 | 91 | 0.255 | 0.0146 | 0.34 |
| Eubacterium | IL-10 | 91 | 0.238 | 0.0228 | 0.3992 |
| Alistipes | TNF-α | 91 | 0.22 | 0.0362 | 0.4425 |
| Alistipes | IL-10 | 91 | 0.21 | 0.0458 | 0.4425 |
| Alistipes | IL-6 | 91 | 0.207 | 0.0488 | 0.4425 |
| Roseburia | IL-10 | 91 | -0.205 | 0.0518 | 0.4425 |
| Roseburia | IL-6 | 91 | -0.2 | 0.0569 | 0.4425 |

**Supplementary Table S3B**. Spearman correlations between differential KEGG orthologs and inflammatory markers in the study population.

| **KO** | **Inflammatory marker** | **N** | **Spearman r** | **Raw P** | **FDR-adjusted P** |
| --- | --- | --- | --- | --- | --- |
| K05808 | IL-18 | 91 | -0.27 | 0.0096 | 0.1828 |
| K10118 | IL-18 | 91 | 0.262 | 0.0121 | 0.1828 |
| K02025 | IL-18 | 91 | 0.26 | 0.0127 | 0.1828 |
| K06871 | IL-18 | 91 | 0.251 | 0.0165 | 0.1828 |
| K10119 | IL-18 | 91 | 0.247 | 0.0182 | 0.1828 |
| K02026 | IL-18 | 91 | 0.244 | 0.0196 | 0.1828 |
| K05808 | TNF-α | 91 | 0.23 | 0.0283 | 0.2268 |

**Note**: Spearman correlation analyses were used to assess associations of differential bacterial taxa or KEGG orthologs with inflammatory markers. Only associations with∣r∣≥0.20 are presented. Raw P represents the unadjusted P value, and FDR-adjusted P represents the Benjamini–Hochberg adjusted P value. No association remained significant after FDR correction.

**Supplementary Table S4. Significantly altered tryptophan-related metabolites in serum identified by systematic re-screening**

| Branch | Metabolite | Level | HMDB ID | Comparison | FC | P value |
| --- | --- | --- | --- | --- | --- | --- |
| Indole | Indolelactic acid | Level 1 | HMDB0000671 | Mod_PSD vs NPSD | 0.6572 | 0.0012 |
| Indole | 3-Indoxyl sulphate | Level 2 | HMDB0000682 | Mod_PSD vs NPSD | 0.8148 | 0.0268 |
| Indole | 4-Indolecarbaldehyde | Level 2 | NA | Mod_PSD vs NPSD | 0.7948 | 0.0068 |
| Indole | Indole-3-acetic acid | Level 2 | HMDB0000197 | Mod_PSD vs NPSD | 0.6193 | 0.0125 |
| Kynurenine | 2-Aminomuconic acid semialdehyde | Level 4 | HMDB0001280 | Mod_PSD vs NPSD | 0.6026 | 0.0136 |
| Precursor | Phenylalanyltryptophan | Level 1 | HMDB0029006 | Mod_PSD vs NPSD | 1.3358 | 0.0142 |

**Note:** Metabolite annotation confidence levels are indicated as Level 1, Level 2, and Level 4. Statistical significance was defined as $p<0.05$in the listed comparison. FC, fold change; HMDB, Human Metabolome Database; Mod, moderate post-stroke depression; NPSD, non-post-stroke depression.

**Supplementary Table S5. Significantly altered tryptophan-related metabolites in feces identified by systematic re-screening**

| Branch | Metabolite | Level | HMDB ID | Comparison | FC | P_value |
| --- | --- | --- | --- | --- | --- | --- |
| Indole branch | 3-Indoxyl sulphate | Level 2 | HMDB0000682 | Mod_PSD vs NPSD | 1.6040 | 0.0082 |
| Kynurenine branch | 6-Phenoxynicotinic acid | Level 2 | NA | Mod_PSD vs NPSD | 0.3569 | 0.0161 |
| Precursor/  biosynthesis | 5-Bromotryptophan | Level 4 | HMDB0240660 | Mod_PSD vs NPSD | 0.8393 | 0.0136 |

Table note: Metabolite annotation confidence levels are indicated as Level 2 and Level 4. Statistical significance was defined as p<0.05 in the listed comparison. FC, fold change; HMDB, Human Metabolome Database; Mod, moderate post-stroke depression; NPSD, non-post-stroke depression.

**Supplementary Table S6. Branch-level summary of evidence for tryptophan metabolism disturbance based on systematic re-screening**

| Branch | Serum findings | Fecal findings | Support level | Interpretation |
| --- | --- | --- | --- | --- |
| Indole branch | Several indole-related metabolites decreased | 3-Indoxyl sulphate increased; additional indole-related trends | Moderate to strong | Predominantly indole-related perturbation |
| Precursor/biosynthesis | Phenylalanyltryptophan increased | 5-Bromotryptophan decreased | Weak to moderate | Suggests upstream alteration |
| Kynurenine branch | Partial changes only | Partial changes only | Weak to moderate | Possibly involved, but not definitive |
| Serotonin branch | No consistent signal | No consistent signal | Weak | Does not support a simple depletion model |
| Other tryptophan-related metabolites | No robust evidence | No robust evidence | Weak | Limited interpretability |

**Note**: Support level reflects the consistency, statistical significance, and biological interpretability of the observed metabolite alterations across serum and fecal datasets, and should not be interpreted as direct evidence of metabolic flux or pathway activation.

**Supplementary Table S7.** FDR-significant Spearman correlations between differential species and differential fecal metabolites after exclusion of exogenous metabolites

| Species | Metabolite | Spearman r | Raw P | FDR-adjusted P |
| --- | --- | --- | --- | --- |
| Bradyrhizobiumsp.000015165 | Desthiobiotin | 0.395 | 0.000109 | 0.0218 |
| Pseudomonas aeruginosa | Daidzein sulfate | 0.365 | 0.000381 | 0.0312 |
| Bradyrhizobiumsp.000015165 | Daidzein sulfate | 0.359 | 0.000469 | 0.0312 |
| Enterococcus_D gallinarum | Desthiobiotin | 0.346 | 0.000779 | 0.0369 |
| Ruminococcus_E sp.003521625 | Epicatechin 3-sulfate | 0.338 | 0.001040 | 0.0369 |
| Enterococcus_B faecium | Desthiobiotin | 0.337 | 0.001167 | 0.0369 |
| Afipia broomeae | Desthiobiotin | 0.327 | 0.001558 | 0.0369 |
| Lachnospira sp. 900772425 | Desthiobiotin | -0.325 | 0.001648 | 0.0369 |
| Duodenibacillus sp. 900767875 | L(-)-Carnitine | 0.323 | 0.001770 | 0.0369 |
| UMGS124 sp. 900539345 | Daidzein sulfate | 0.322 | 0.001844 | 0.0369 |
| Ruminococcus_E sp. 003521625 | Desthiobiotin | -0.319 | 0.002057 | 0.0374 |

**Note**: Spearman correlation analysis was performed to evaluate pairwise associations between differential species and differential fecal metabolites. Raw P values were adjusted for multiple comparisons using the Benjamini–Hochberg false discovery rate (FDR) method. To reduce potential interference from drugs, chemical reagents, and other exogenous exposure-related compounds, explicitly exogenous metabolites were excluded during result curation and data visualization. This table presents only the associations that remained statistically significant after FDR correction following exclusion of exogenous metabolites.

**Supplementary Table S8**. Representative KO–metabolite correlations corresponding to Figure 12B

| KO | Metabolite | Spearman r | Raw P | FDR-adjusted P | Category |
| --- | --- | --- | --- | --- | --- |
| K20381 | Desthiobiotin | 0.403 | 0.000075 | 0.015 | FDR-significant |
| K21834 | Gentisic acid | -0.318 | 0.0022 | 0.216 | Trend (negative) |
| K05808 | 11-Epiprostaglandin E1 | -0.289 | 0.0056 | 0.336 | Trend (negative) |
| K02027 | N-Acetylhistidine | 0.261 | 0.0127 | 0.336 | Trend (positive) |
| K02529 | L(-)-Carnitine | 0.249 | 0.0177 | 0.336 | Trend (positive) |
| K01804 | Epicatechin 3-sulfate | -0.236 | 0.0248 | 0.354 | Trend (negative) |

**Note:** This table summarizes representative KO–metabolite associations shown in Figure 12B after removal of explicitly exogenous metabolites. Spearman correlation analysis was performed between differential KOs and differential fecal metabolites, and raw P values were adjusted using the Benjamini–Hochberg false discovery rate method.

**Supplementary Table S9**：Spearman correlation coefficients, raw P values, and Benjamini-Hochberg adjusted P values between differential gut microbial species and selected serum metabolites

| Species | Metabolite | Spearman r | Raw P | FDR-adjusted P |
| --- | --- | --- | --- | --- |
| Pseudomonas aeruginosa | LysoPI(16:0/0:0) | -0.314 | 0.0024 | 0.0508 |
| Lachnospira sp.900772425 | LysoPI(16:0/0:0) | 0.294 | 0.0046 | 0.0835 |
| Lachnospira eligens_A | Gamma-Caprolactone | -0.284 | 0.0065 | 0.0924 |
| HGM12814 sp. 900770165 | Gamma-Caprolactone | -0.279 | 0.0075 | 0.0934 |
| Ruminococcus_G gauvreauii | NeuNGc | 0.280 | 0.0074 | 0.0934 |
| Campylobacter_A concisus_P | Gamma-Caprolactone | -0.276 | 0.0082 | 0.0966 |
| Olsenella_E sp. 900540955 | LysoPI(16:0/0:0) | -0.258 | 0.0136 | 0.1305 |
| Ruminococcus_E sp. 003521625 | Gamma-Caprolactone | -0.255 | 0.0145 | 0.1305 |
| Bradyrhizobium sp. 000015165 | Gamma-Caprolactone | 0.256 | 0.0144 | 0.1305 |
| Eggerthella sp. 014287365 | Gamma-Caprolactone | 0.256 | 0.0147 | 0.1305 |
| Lachnospira rogosae_A | Gamma-Caprolactone | -0.240 | 0.0222 | 0.1707 |
| Ruminococcus_E sp. 003521625 | LysoPI(16:0/0:0) | 0.238 | 0.0233 | 0.1755 |
| Lachnospira rogosae_A | LysoPI(16:0/0:0) | 0.236 | 0.0246 | 0.1755 |
| Pseudomonas aeruginosa | PE(16:0/20:4(5Z,8Z,11Z,14Z)) | 0.233 | 0.0260 | 0.1755 |
| Duodenibacillus sp. 900767875 | Gamma-Caprolactone | 0.232 | 0.0272 | 0.1755 |
| Lachnospira sp. 000436535 | PE(16:0/20:4(5Z,8Z,11Z,14Z)) | -0.232 | 0.0274 | 0.1755 |
| Duodenibacillus sp. 900767875 | LysoPI(16:0/0:0) | -0.231 | 0.0276 | 0.1755 |
| Lachnospira rogosae_A | PE(16:0/20:4(5Z,8Z,11Z,14Z)) | -0.218 | 0.0378 | 0.2085 |
| Olsenella_B sp. 000752675 | LysoPI(16:0/0:0) | -0.213 | 0.0429 | 0.2231 |
| Lachnospira sp. 003537285 | Gamma-Caprolactone | -0.210 | 0.0462 | 0.2335 |

**Note**: Spearman correlation analysis was performed to evaluate pairwise associations between differential gut microbial species and selected serum metabolites. Raw P values were adjusted for multiple comparisons using the Benjamini–Hochberg false discovery rate method. This table presents representative correlation results supporting the trends described in the main text. None of the tested associations remained statistically significant after FDR correction.

**Supplementary Table S10.** Spearman correlation results between differential functional genes and selected serum metabolites

| KO | Metabolite | Spearman r | Raw P | FDR-adjusted P |
| --- | --- | --- | --- | --- |
| K06331 | LysoPI(16:0/0:0) | 0.378 | 0.0002 | 0.0598 |
| K11232 | LysoPI(16:0/0:0) | 0.275 | 0.0084 | 0.1484 |
| K25142 | Gamma-Caprolactone | 0.323 | 0.0019 | 0.0678 |
| K02529 | Gamma-Caprolactone | 0.298 | 0.0043 | 0.1096 |
| K01804 | Gamma-Caprolactone | 0.256 | 0.0147 | 0.1726 |
| K06331 | Gamma-Caprolactone | -0.267 | 0.0106 | 0.1624 |
| K05808 | Gamma-Caprolactone | -0.309 | 0.0030 | 0.0847 |
| K03409 | Gamma-Caprolactone | -0.248 | 0.0178 | 0.1926 |
| K03409 | LysoPI(16:0/0:0) | 0.236 | 0.0243 | 0.2336 |
| K10548 | LysoPI(16:0/0:0) | -0.214 | 0.0417 | 0.2873 |
| K10548 | Gamma-Caprolactone | 0.216 | 0.0400 | 0.2860 |
| K01239 | Gamma-Caprolactone | 0.221 | 0.0353 | 0.2666 |
| K00853 | Gamma-Caprolactone | 0.213 | 0.0424 | 0.2873 |
| K03409 | 3-Fluoro-2-oxopropanoic acid | -0.224 | 0.0330 | 0.2666 |
| K06331 | 3-Fluoro-2-oxopropanoic acid | -0.230 | 0.0282 | 0.2503 |

**Note**: Spearman correlation analysis was performed to evaluate pairwise associations between differential functional genes and selected serum metabolites. Raw P values were adjusted for multiple comparisons using the Benjamini–Hochberg false discovery rate method. This table presents representative correlation results supporting the trends described in the main text. None of the tested associations remained statistically significant after FDR correction.

**Supplementary Figure：**

**Supplementary Figure S1**：Network topology analysis of representative differential metabolites in fecal and serum metabolomic comparisons related to PSD

**Note**：(A) Metabolite–enzyme–pathway association network constructed from representative differential fecal metabolites identified in the comparison between the Mild-PSD group and the NPSD group.(B) Metabolite–enzyme–pathway association network constructed from representative differential fecal metabolites identified in the comparison between the Mod-PSD group and the NPSD group.(C) Metabolite–enzyme–pathway association network constructed from representative differential serum metabolites identified in the comparison between the Mild-PSD group and the Mod-PSD group.

In the networks, circles represent pathways, squares represent metabolites, and triangles represent enzymes. Edges indicate annotated associations supported by database information. These networks are provided to illustrate the connection patterns among representative differential metabolites, related enzymes, and enriched pathways in different metabolomic comparisons.

**Supplementary Figure S2**：ROC analysis of representative serum differential metabolites for discriminating PSD severity

**Note**：Representative ROC curves of serum differential metabolites for group discrimination in PSD-related comparisons. Left, PI(18:2/0:0) for distinguishing Mild-PSD from NPSD (AUC = 0.732); middle, 5alpha-androstan-3alpha,17alpha-diol monosulfate for distinguishing Mod-PSD from NPSD (AUC = 0.796); right, 2-Aminomuconic acid semialdehyde for distinguishing Mild-PSD from Mod-PSD (AUC = 0.795). The x-axis indicates 1-specificity, and the y-axis indicates sensitivity. These plots are provided as supplementary results for the evaluation of the discriminatory performance of representative serum metabolites.

**References**

1. Liu H, Zhu X, Wang J, et al. Mechanisms and therapeutic potential of the cGAS–STING signaling pathway in macrophage activation and polarizationcGAS–STING. Biochemical Pharmacology. 2026;250:118031.
2. Wang Y, Zhang J, Wang J, Zhang Y, Sun J, Gong J, Yao X, Sun H. The Dual Role of Interleukin-6 in the Pathophysiology of Skeletal Muscle: Mechanisms, Challenges, and Therapeutic Prospects. Pharmaceuticals 2026; 19:868.
3. Shen D, Zhao Z, Ji Y, Zhang J, Yao X, Qi L, Sun H. The role of ferroptosis in epilepsy: Mechanisms and targeted therapies. Biochemical Pharmacology 2026; 249:117912.
4. Qi L, Yi J, Ji Y, Jiang H, Sun J, Shen Y, Sun H. Network of cell death in myocardial ischemia-reperfusion injury: Mechanisms and targeted therapeutic strategies. Biochemical Pharmacology 2026; 250:117999.
5. Chen J, Liu B, Yao X, Yang X, Sun J, Yi J, Xue F, Zhang J, Shen Y, Chen B, et al. AMPK/SIRT1/PGC‐1α signaling pathway: Molecular mechanisms and targeted strategies from energy homeostasis regulation to disease therapy. CNS Neurosci Ther 2025; 31:e70657.
6. Yan Y, Li M, Lin J, Ji Y, Wang K, Yan D, Shen Y, Wang W, Huang Z, Jiang H, et al. Adenosine monophosphate activated protein kinase contributes to skeletal muscle health through the control of mitochondrial function. Front Pharmacol 2022; 13:947387.
